# A Systematic Review of Lifestyle Interventions for Neuropathy and Neuropathic Pain: Alcohol Consumption and Avoidance

**DOI:** 10.3390/brainsci16060551

**Published:** 2026-05-22

**Authors:** Michael Klowak, Ezra J. Bado, Aquilla Reid-John, Rumaysa Dawood, Candice Madakadze, Andrea K. Boggild

**Affiliations:** 1 Institute of Medical Science, University of Toronto, Toronto, ON M5S 1A1, Canada; 2 Tropical Disease Unit, Toronto General Hospital, Toronto, ON M5G 2C4, Canada; 3 Department of Medicine, University of Toronto, Toronto, ON M5S 3H2, Canada

**Keywords:** lifestyle interventions, neuropathic pain, neuropathy, alcohol, alcohol dependence, avoiding risky substances, systematic review

## Abstract

**Highlights:**

**What are the main findings?**

- Alcohol consumption and dependence were associated with increased incidence, prevalence, and/or severity of neuropathy/neuropathic pain across observational studies, with over half of included studies demonstrating statistically significant positive associations.
- Pooled analysis showed higher odds of neuropathy with alcohol exposure (consumption: OR 1.29 95% CI [1.10–1.50]; dependence: 1.24 [1.16–1.33]), while longer duration and greater exposure were linked to increased severity and diminished electrophysiological function, and abstinence was associated with clinical improvement.

**What are the implications of the main findings?**

- Findings support a potential contributory role of alcohol use, particularly dependence and chronic exposure, in the development and progression of neuropathy/neuropathic pain, consistent with known neurotoxic and metabolic mechanisms.
- Alcohol abstinence and reduction may represent a low-risk, low-cost, adjunctive strategy for improving neuropathy/neuropathic pain outcomes, although causal inference is limited by heterogeneity and very low certainty of evidence, highlighting the need for robust interventional studies.

**Abstract:**

**Background**: Neuropathy and neuropathic pain (NP) are globally prevalent, remain difficult to manage, and are often exacerbated by underlying lifestyle factors. Alcohol use, particularly in the context of chronic consumption or dependence, is a recognized contributor to peripheral nerve damage, yet its association with neuropathy/NP has not been systematically evaluated. This systematic review synthesizes the current evidence on alcohol exposure, including quantity, frequency, and dependency, and its association with the incidence, prevalence, and severity of neuropathy/NP. **Methods**: This systematic review included observational studies assessing alcohol consumption patterns or dependence in relation to neuropathy/NP outcomes and was conducted in accordance with PRISMA guidelines. Exposure types were analyzed independently, and pooled odds ratios and relative risks were generated when sufficient data were available. The review was registered with PROSPERO number CRD42023484158. **Results**: Following de-duplication and exclusions, 76 studies were included, comprising cohort (n = 15), case–control (n = 12), and cross-sectional (n = 49) designs. While associations varied by study design and exposure category, alcohol dependence and consumption were more consistently linked with increased neuropathy incidence and severity, including electrophysiological evidence of compromised function. Notably, in studies examining alcohol cessation, abstinence was linked to clinical improvements in neuropathy/NP symptoms such as hypoesthesia and muscle weakness. While heterogeneity and risk of bias were present, largely due to the subjective classification of alcohol exposure and a lack of universally applied objective neuropathy measurement tools, multiple pooled estimates reached statistical significance. **Conclusions**: Evidence from observational studies supports an association between alcohol use, especially dependence, and the development and progression of neuropathy/NP, although causality remains unproven. Abstinence may offer therapeutic benefit, though further abstinence- and/or harm reduction-related interventional studies are required to clarify causality and guide low-cost, adjunctive strategies for alcohol-related neuropathy/NP.

## 1. Introduction

Neuropathy and neuropathic pain (NP), caused by lesions or diseases of the nervous system, are conditions of increasing global relevance, with up to 10% of individuals worldwide suffering from their debilitating effects [1]. Alcohol-related neuropathy remains a significant contributor to this burden, as recent estimates indicate it may account for 8–10% of polyneuropathies across all populations [2]. Patients with alcohol use disorders (AUDs) already experience substantial morbidity, due in part to alcohol-related neurological disability, further complicating its impact on the overall healthcare system [3]. Comprehensive systematic reviews and epidemiological data suggest that neuropathy/NP conditions are prevalent in up to 66% of all individuals with chronic alcohol use disorders [2,3]. As a result, this relationship underscores the significant and persistent social and financial burden that neuropathy/NP exerts at an individual and population level. It is estimated that costs associated with harmful alcohol use, including alcohol-related neuropathies, account for roughly 1–2% of the global GDP [4]. Given this substantial negative physical and economic impact, there is a dire need for supportive therapies that more effectively address the symptomatology of alcohol-related neuropathies.

The neuropathophysiology associated with chronic and excessive alcohol use is highly insidious, characterized by slow and progressive neurological degeneration [5]. Over the course of months to years, alcohol users develop distal pain, numbness, diminished reflexes, impaired vibration perception, and motor/sensory electrophysiological abnormalities, typically affecting the lower and upper limbs [2,6]. The pathogenesis of alcohol-related neuropathy/NP involves both direct and indirect mechanisms. Indirectly, chronic and excessive alcohol consumption contribute to nutritional deficiencies, including reducing hepatic storage of thiamine, limiting phosphorylation, and impairing absorption. These metabolic disruptions, compounded by the poor caloric intake commonly associated with alcohol use disorders, ultimately lead to peripheral nervous system sensitization and degeneration [5,6]. Likewise, excessive ethanol metabolism produces reactive oxygen species and other cytotoxic byproducts that significantly damage and impair nerve cells. Therefore, the direct effects of ethanol consumption further contribute to peripheral nerve injury, leading to primary axonal damage and secondary demyelination of motor and sensory fibers [5,6]. Emerging evidence also suggests that alcohol may alter microRNA expression, thereby influencing neural signaling involved in pain processing, with growing interest in their role as biomarkers in neuropathic conditions [7,8]. While these mechanisms represent the primary pathways identified to date, preclinical studies continue to investigate additional molecular and neuroimmune processes [5]. Despite this, effective management of neuropathic symptoms remains limited, and additional adjunctive treatment strategies targeting these underlying mechanisms are needed.

Conventional pharmacologic therapies for neuropathy/NP, including antidepressants, anticonvulsants, and opioids, remain only somewhat effective, typically providing less than 30% pain relief [1]. In the context of alcohol-related neuropathy/NP, additional nutritional support and adjunctive therapies have also been explored beyond gold standard pharmaceuticals. Vitamin supplementation, including thiamine, B6, and B12, as well as antioxidant compounds such as alpha-lipoic acid and N-acetylcysteine may confer protective effects, limiting overall morbidity [2,5,6]. Likewise, lifestyle interventions and/or modifications, including domains such as nutrition, physical activity, stress management, sleep, social connectedness, and avoidance of harmful substances (e.g., tobacco and alcohol), have recently emerged as low-risk, cost-effective strategies for reducing the overall morbidity of neuropathy/NP due to various etiologies [9]. Specifically, modification of alcohol consumption, including reduction or abstinence, has been shown to successfully manage neuropathy and NP symptoms; however, evidence remains limited given the insidious nature of alcohol dependence and addiction [2]. As part of an ongoing systematic review series, we have examined individual lifestyle domains independently in relation to neuropathy/NP, including diet and tobacco use [2,9,10]. Building on this framework, the present review specifically seeks to assess how alcohol consumption (including frequency, quantity, and duration) impacts the incidence, prevalence, and severity of neuropathy and NP. Ultimately, it is hypothesized that alcohol use meaningfully contributes to disease burden, and that cessation may help reduce overall morbidity.

## 2. Materials and Methods

The “Preferred Reporting Items for Systematic Reviews and Meta-Analysis” (PRISMA) guidelines/checklist were followed (Table S1), and the review was prospectively registered with the International Prospective Register of Systematic Reviews (PROSPERO: CRD42023484158). Interventional and observational studies, including cohort, case–control, and cross-sectional designs, were included if they assessed lifestyle variables as interventions, exposures, outcomes, or stratification factors in populations with neuropathy and/or neuropathic pain, regardless of etiology. The search spanned from database inception to 21 October 2025. This work is part of a larger systematic review series on lifestyle interventions and neuropathy/neuropathic pain and specifically evaluates alcohol consumption, dependence, and related exposures. Detailed methodology for the overarching systematic review series, including full eligibility criteria, outcome measures, data sources, search strategy, study selection, data extraction, statistical analysis, risk of bias and certainty of evidence assessments, and meta-analyses, have been previously reported and are available in open-access format [9,11].

In brief, the following comprehensive search strategy was applied across multiple databases (including Medline, PubMed, Scopus, Embase, and LILACS): (neuropathic pain OR neuropathy OR neuritis OR diabetic neuropathy OR peripheral neuropathy OR chemical neuropathy OR toxic neuropathy OR chemotherapy-induced peripheral neuropathy OR vitamin B deficiency) AND (nutrition OR nutrient OR nutritionally compromised OR micronutrient OR macronutrient OR malnutrition OR nutritional status OR nutrient supplement* OR plant based OR vegetarian OR vegan OR mediterranean diet OR diet OR physical activity OR exercise OR lifestyle OR lifestyle interventions OR BMI OR smoking OR alcohol OR stress OR sleep). Records were independently screened by two reviewers using Covidence. Eligibility was defined using a PICO-based framework, including individuals with neuropathy or NP of any etiology, with alcohol-related exposures assessed against non-exposed groups, and outcomes encompassing incidence, prevalence, and severity. From included studies, data were extracted on study characteristics, exposure definitions, and neuropathic outcomes at the highest level of detail reported, enabling pooled estimates to be calculated where appropriate. Study quality was then appraised using Joanna Briggs Institute-based risk of bias tools, alongside evaluation of certainty of evidence using the GRADE (“Grading of Recommendations, Assessment, Development, and Evaluation”) framework. These data were subsequently synthesized through meta-analyses using stratified exposure categories, with odds ratios and relative risks calculated and pooled where feasible.

### Statistical and Meta-Analyses

Alcohol exposure was stratified to capture differences in reported consumption patterns and dependency, including consumption status (never, former, current, or any use/history of use), dependency-related measures (addiction, AUD, abuse, or excessive consumption), and guideline-based thresholds (e.g., >30 g/day or exceeding sex-specific recommendations such as >7/14 or >14/21 units for females/males). Given anticipated heterogeneity in exposure definitions, classifications ranged from simple dichotomous measures (e.g., consumption yes/no) to more detailed categorizations and broad quantitative estimates, including intake reported as drinks, grams, or units per day or week, with variability in how thresholds and units were defined across studies. Where exposure categories combined former, current, lifetime, or unspecified alcohol use, these classifications were retained as reported, but interpreted as broad exposure categories, as timing of exposure could not be consistently distinguished across studies. Some exposure categories were defined broadly enough to include individuals with prior alcohol use, potentially leading to misclassification, and adding variability to pooled results. To mitigate this, data were captured at the most detailed level available from each study to enable more refined stratified analyses.

Although included studies specified neuropathy subtypes and underlying etiologies, there was insufficient overlap within individual etiologic subgroups to support meaningful subgroup-specific pooled analyses. As such, neuropathy and NP outcomes were pooled within study design and exposure strata to preserve analytic stability, while etiologic heterogeneity was considered during interpretation and certainty of evidence assessment. Studies enrolling alcohol-dependent populations or individuals with alcohol-related neuropathy were retained, as they contributed relevant data on neuropathy/NP incidence, prevalence, severity, electrophysiological impairment, and response to abstinence, but were interpreted within the context of their underlying study populations and exposure definitions, and not as independent estimates of alcohol as a causal risk factor. Pooled estimates were generated only when studies of the same design reported sufficiently comparable alcohol exposure classifications and neuropathic outcomes. Given the observational nature of the included literature, particularly among cross-sectional studies, temporality between alcohol exposure and neuropathy/NP outcomes could not always be established. Accordingly, pooled estimates reflect associations across heterogenous populations and study designs rather than causal effect estimates, and these methodological constraints were incorporated into risk of bias and certainty of evidence assessments using the GRADE framework to inform interpretation of the synthesized results.

## 3. Results

### 3.1. Literature Search

**Figure 1:**
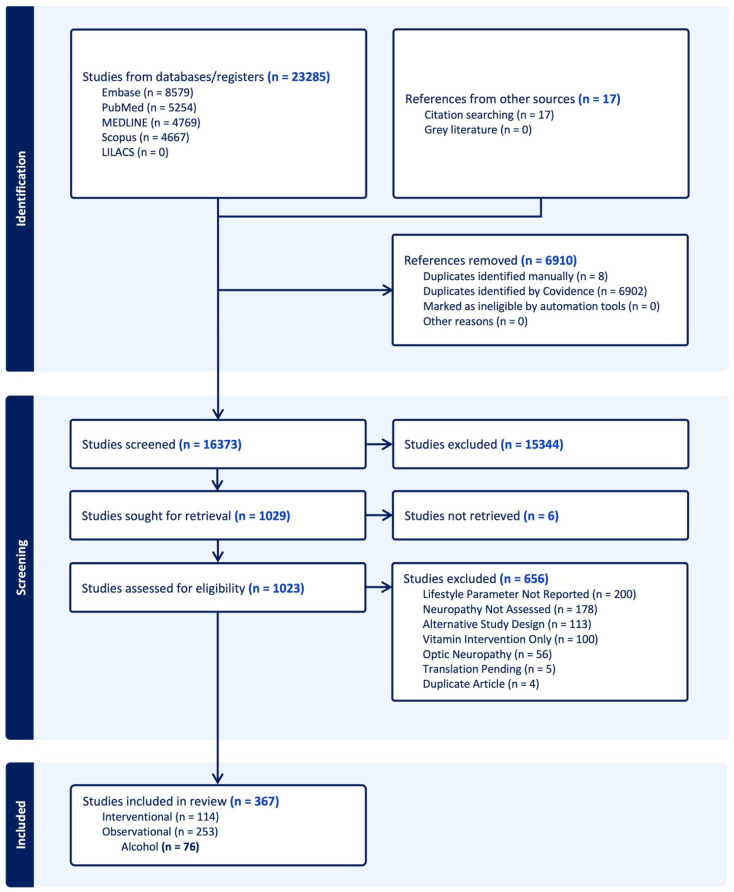
PRISMA flowchart.

The search strategy identified 23,285 articles across five databases, including Embase (8579), PubMed (5254), Medline (4769), Scopus (4667), and LILACS (0), with 17 additional articles captured via bibliography screening. Following deduplication, 16,373 records remained, of which 1023 full-text articles were assessed for final inclusion. A total of 367 records described primary outcomes relevant to the systematic review series, of which 76 reported alcohol use in the context of neuropathy or neuropathic pain (Table 1). A comprehensive summary of the screening process and reasons for article exclusion is available in Figure 1.

### 3.2. Included Studies

Records identified in this systematic review were all observational in nature. Fifteen cohort studies were included, comprising 252,171 participants, with participant numbers ranging from 10 to 222,334 per study [12,13,14,15,16,17,18,19,20,21,22,23,24,25,26]. Participant ages ranged from 18 to 90 years across the cohort studies, with females comprising 48.44% of the collective sample. Most studies were conducted in high-income settings, with the exception of one study from Zimbabwe, as classified by the World Bank [23]. Publication years spanned 1982 to 2025. The majority of the cohort studies investigated populations with peripheral neuropathy (PN) (7) [13,15,17,18,20,25,26], or broadly defined neuropathy (6) [12,19,21,23,24], with fewer examining polyneuropathy (PoN) (2) [14,16], and complex regional pain syndrome (CRPS) (1) [22]. Neuropathy etiologies were predominantly diabetes mellitus (7) [12,13,14,15,19,21,24], cancer (4) [20,23,25,26], and alcohol dependence (3) [17,18,19], as well as trauma and HIV (1 each) [16,22]. Cohort studies all reported neuropathy status in relation to alcohol exposure, categorized as any consumption (7) [13,15,20,22,23,25,26], alcohol dependence (6) [12,16,17,18,19,21], or consumption by guidelines (2) [14,24]. However, only eight studies [12,13,14,19,21,22,24,25] provided sufficient data to calculate odds ratios and relative risks for neuropathy incidence among exposed versus non-exposed groups.

Across 12 case–control studies, 1481 cases and 2441 controls were analyzed, with sample sizes spanning 18 to 1156 individuals per study [27,28,29,30,31,32,33,34,35,36,37,38]. The mean age ranged from 27 to 77 years, with females comprising 45.9% of total participants. Publication date ranged from 1970 to 2023, and most studies were conducted in high-income countries, aside from one from Ethiopia [32]. PN (4) [27,32,36,38] and ulnar neuropathy of the elbow (3) [31,35,37] were the most commonly studied conditions, with additional cases of chronic inflammatory demyelinating polyradiculoneuropathy (CIDP), small fiber neuropathy (SFN), distal symmetric neuropathy (DSN), PoN, and general neuropathy (1 each) [28,29,30,33,34], attributed to any etiology (5) [28,29,31,35,37], alcohol dependence (4) [27,33,36,38], or diabetes mellitus (3) [30,32,34]. Alcohol exposure was categorized as any consumption (7) [28,30,31,32,34,35,37] and alcohol dependence (5) [27,29,33,36,38]. Six studies [28,29,30,32,35,36] reported sufficient data to calculate odds ratios for the association between alcohol exposure and neuropathy.

Forty-nine cross-sectional studies were included, encompassing 90,889 participants, with study sizes ranging from 18 to 25,710 individuals [39,40,41,42,43,44,45,46,47,48,49,50,51,52,53,54,55,56,57,58,59,60,61,62,63,64,65,66,67,68,69,70,71,72,73,74,75,76,77,78,79,80,81,82,83,84,85,86,87]. Participant ages spanned 15 to 100 years, with females accounting for 43.75% across studies. One ecological cross-sectional study evaluated insurance reimbursement claims per 1000 population, covering up to 126 million individuals, and was excluded from these averages [68]. Publications spanned 1965 to 2024 and included both high- (43/49) [39,40,41,42,43,44,45,46,48,50,51,52,53,54,55,56,57,59,60,61,62,63,64,65,66,67,68,69,70,71,72,76,77,78,79,80,81,82,83,84,85,86,87] and low-income (6/49) [47,49,58,73,74,75] countries. PN (29) [39,40,41,46,47,48,49,52,53,54,57,59,61,62,64,66,67,70,71,72,73,74,75,76,77,78,80,83,85] accounted for over half of the diagnoses across all studies, followed by PoN (8) [44,50,56,63,79,81,84,86], painful neuropathy (6) [42,51,60,65,68,82], and general neuropathy (6) [43,45,55,58,69,87] to a lesser extent. Alcohol dependence (22) [40,41,44,47,50,52,53,54,61,64,66,67,69,71,74,78,79,81,82,85,86,87] and diabetes mellitus (14) [39,43,46,49,56,57,58,59,60,62,73,77,80,84] were the most frequently reported etiologies, followed by any cause (8) [42,48,51,55,63,65,68,83], cancer (4) [70,72,75,76], and BMI (1) [45]. Alcohol exposure was categorized as any consumption (25) [39,42,43,45,46,48,49,51,54,57,58,59,62,63,64,68,70,72,73,74,75,76,81,83,84], alcohol dependence (22) [40,41,44,47,50,52,53,55,60,61,65,66,67,69,71,78,79,80,82,85,86,87], and overconsumption by guidelines (2) [56,77]. Prevalence estimates of neuropathy were reported in relation to alcohol exposure in 17 [39,42,43,46,48,51,56,57,58,59,67,70,73,77,80,84,85] studies, with odds ratios used to quantify the strength of these associations.

### 3.3. Risk of Bias

**Figure 2:**
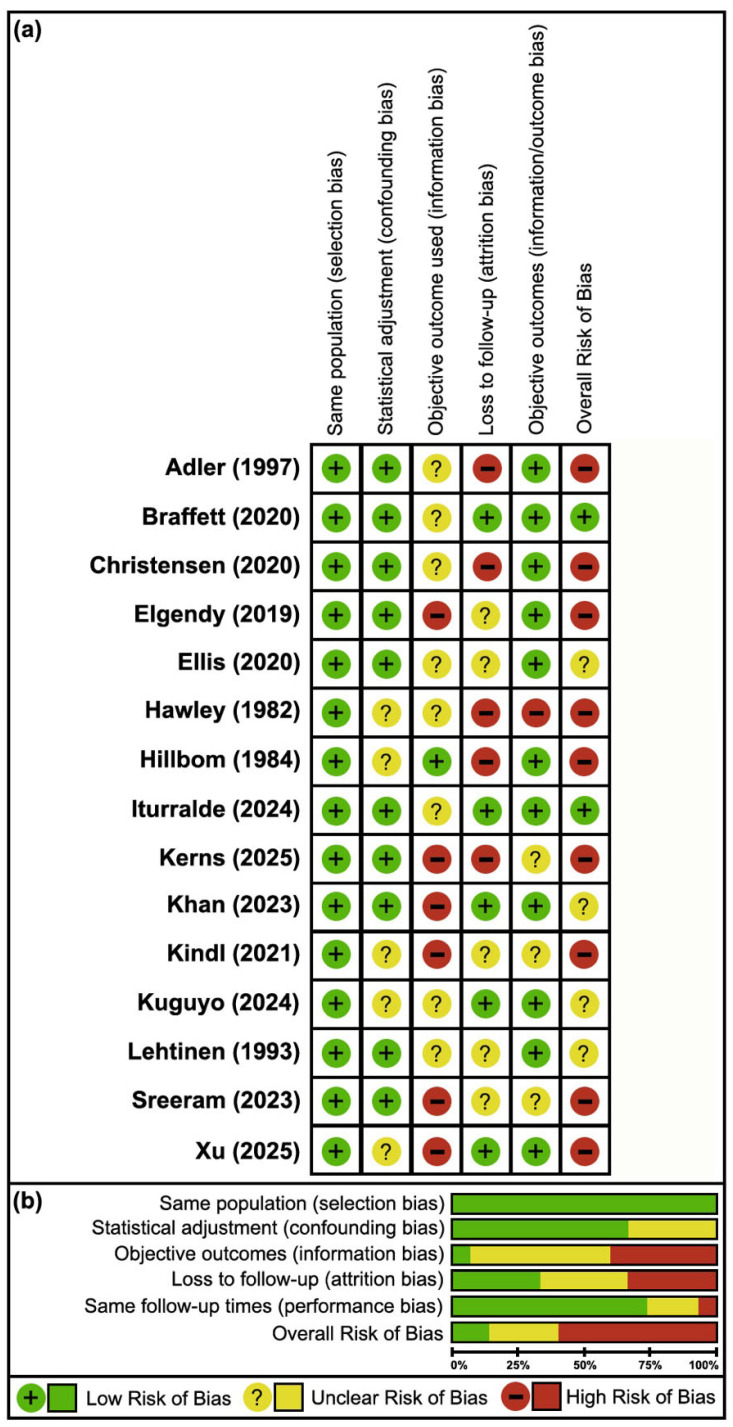
Risk of bias assessment for cohort studies. (**a**) Risk of bias summary by cohort study [12,13,14,15,16,17,18,19,20,21,22,23,24,25,26]; (**b**) summary of risk of bias items by bias item.

Risk of bias among cohort studies was predominantly moderate, with low risk identified in 56% (42/75) of assessed measures. Information and attrition biases were most prominent, as most studies relied on non-objective outcomes (14/15, 93%) [12,13,14,15,16,17,19,20,21,22,23,24,25,26] and reported moderate to high loss to follow-ups (10/15, 67%) [12,14,15,16,17,18,20,22,24,25], while selection, confounding, and outcome biases were generally low (0/15, 0%; 5/15, 33% [17,18,22,23,26]; 4/15, 27% [17,20,22,25], respectively) (Figure 2).

**Figure 3:**
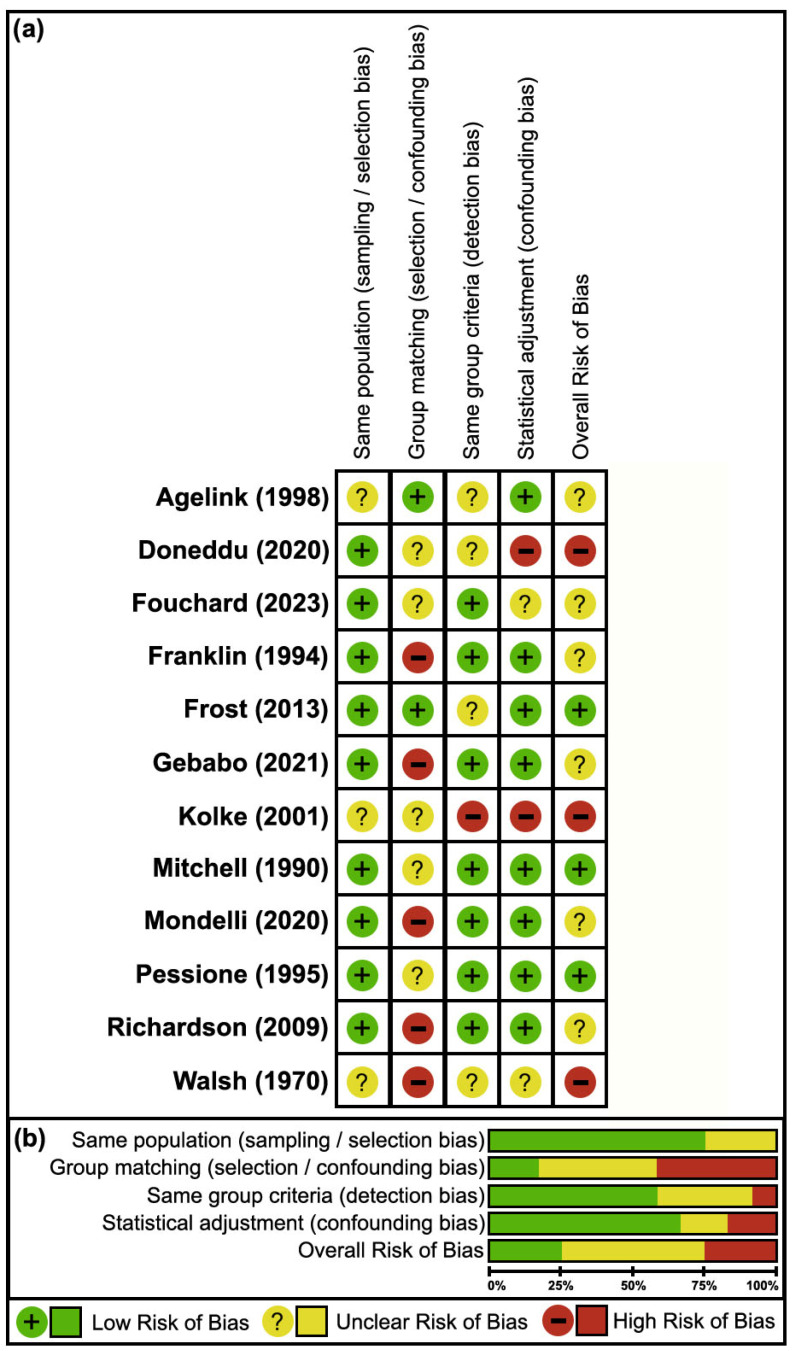
Risk of bias assessment for case–control studies. (**a**) Risk of bias summary by case–control study [27,28,29,30,31,32,33,34,35,36,37,38]; (**b**) summary of risk of bias items by bias item.

Case–control studies demonstrated an overall moderate risk of bias, with 54% (26/48) of domains rated as low-risk. Poor matching of cases and controls represented the most prominent limitation (10/12, 83%) [28,29,30,32,33,34,35,36,37,38], while sampling/selection, detection, and confounding biases were less prevalent (3/12, 25% [27,33,38]; 5/12, 42% [27,28,31,33,38]; 4/12, 33% [28,29,33,38], respectively) (Figure 3).

**Figure 4:**
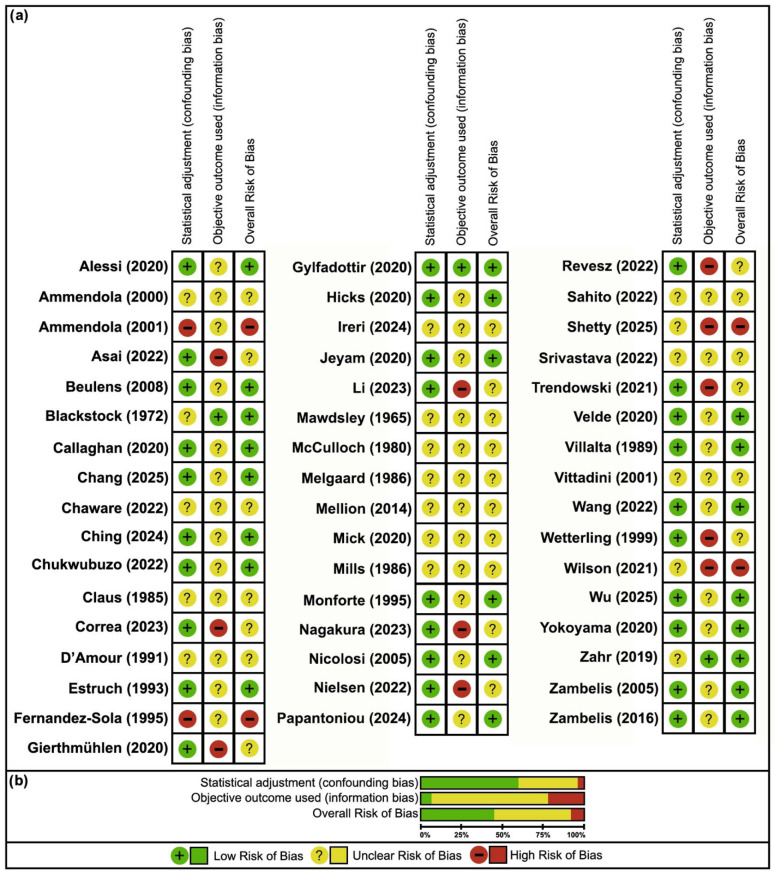
Risk of bias assessment for cross-sectional studies. (**a**) Risk of bias summary by cross-sectional study [39,40,41,42,43,44,45,46,47,48,49,50,51,52,53,54,55,56,57,58,59,60,61,62,63,64,65,66,67,68,69,70,71,72,73,74,75,76,77,78,79,80,81,82,83,84,85,86,87]; (**b**) summary of risk of bias items by bias item.

Comparatively, cross-sectional studies were associated with a higher risk of bias overall, with just 33% (32/98, 33%) of assessed domains classified as low-risk. Statistical adjustments were adequate in most studies (29/49, 59%) [39,42,43,45,46,48,49,51,53,55,56,57,59,60,67,68,69,70,71,72,76,77,78,80,81,83,84,86,87]; however, information bias remained a major concern due to the widespread use of non-objective outcomes (46/49, 94%) [39,40,41,42,43,45,46,47,48,49,50,51,52,53,54,55,57,58,59,60,61,62,63,64,65,66,67,68,69,70,71,72,73,74,75,76,77,78,79,80,81,82,83,84,86,87]. Only one study [56] achieved an overall low risk of bias, whereas the vast majority (75/76, 99%) had at least one domain classified as unclear or high risk (Figure 4).

### 3.4. Summary of Findings

**Figure 5:**
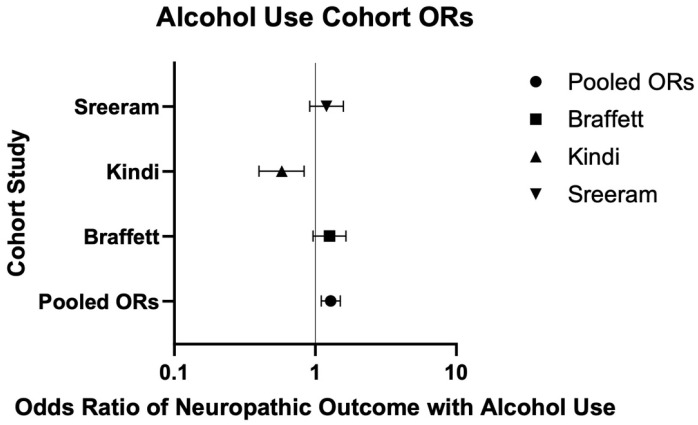
Forest plot of odds ratios of neuropathic outcome according to alcohol consumption in cohort studies.

Quantitative and qualitative synthesis of neuropathy and NP incidence, prevalence, and association with alcohol consumption is available in Table 1 and Table 2. Of the seven cohort studies assessing alcohol consumption and neuropathy/NP, only one (14%) reported a significant positive association between alcohol consumption and higher pain intensity [22] (Table 1). Five studies (71%) found no significant association [13,20,23,25,26], and one study (14%) reported significantly lower odds of neuropathy with more frequent alcohol consumption [15] (Table 1). Collectively, three studies (43%) provided sufficient data for pooled analysis. Among these, the pooled odds of a neuropathic outcome in those exposed vs. unexposed (i.e., odds ratio) was 1.29 [95% confidence interval 1.10–1.50], indicating 29% higher odds of incident neuropathy, while the pooled relative risk was 1.14 [1.05–1.23] [13,22,25] (Table 2, and Figure 5).

**Figure 6:**
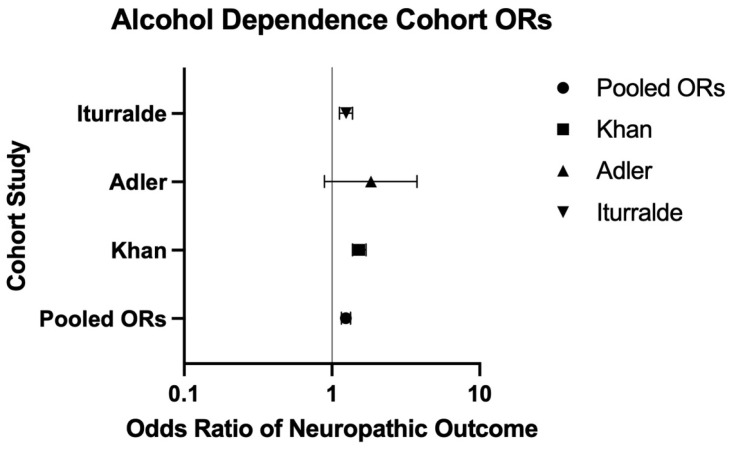
Forest plot of odds ratios of neuropathic outcome according to alcohol dependence in cohort studies.

Of the six cohort studies assessing alcohol dependence and neuropathy/NP, four (67%) reported a significant positive association, either with increased odds of incident neuropathy [12,19], or with clinical and electrophysiological improvement with alcohol abstinence [17,18] (Table 1). The remaining two studies (33%) did not report a significant association [16,21] (Table 1). Overall, three studies (50%) provided sufficient data for pooled analysis. Among these, the pooled odds ratio was 1.24 [1.16–1.33], and the pooled relative risk was 1.19 [1.12–1.25], suggesting that individuals with AUD, or who were dependent on alcohol, had 24% higher odds of incident neuropathy compared to non-dependent or abstinent individuals [12,19,21] (Table 2, and Figure 6).

**Figure 7:**
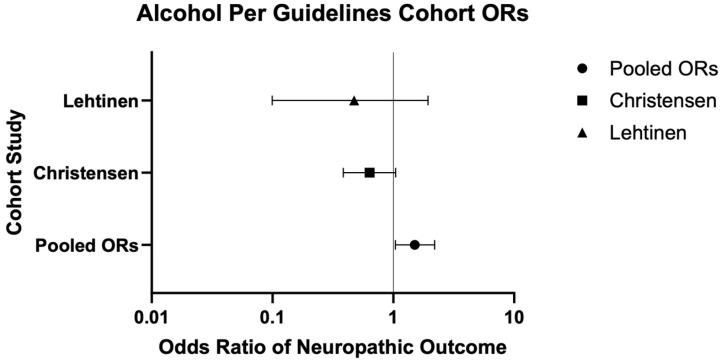
Forest plot of odds ratios of neuropathic outcome according to alcohol overconsumption as per guidelines in cohort studies [14,24].

The remaining cohort studies focused on alcohol overconsumption by specific guidelines. Alcohol consumption in excess of guideline recommendations (that is, of >14/21 units per week for females/males) was significantly associated with increased prevalent NP [14], while consumption >30 g per week was not [24] (Table 1). The calculated odds ratio was 1.51 [1.04–2.2], indicating a 51% increased risk of neurophysiological deterioration and pain in those exceeding thresholds compared to those who are not. The calculated relative risk was 1.31 [1.02–1.71] (Table 2 and Figure 7).

**Figure 8:**
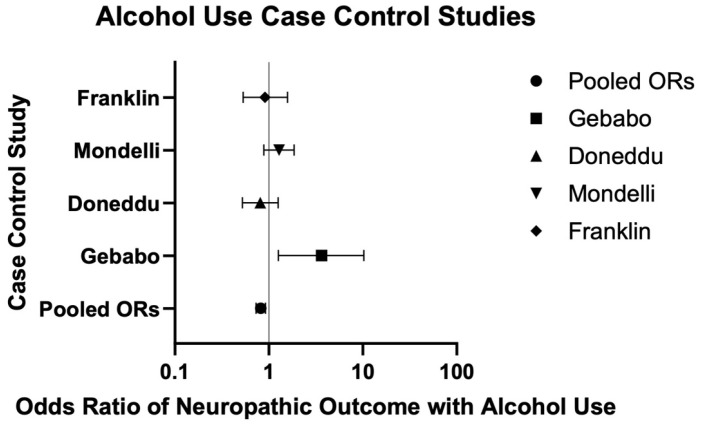
Forest plot of odds ratios of neuropathic outcome according to alcohol consumption in case–control studies [28,30,32,35].

Seven case–control studies assessed alcohol consumption and neuropathy/NP, of which only one study [32] (14%) reported significantly greater alcohol consumption in cases versus controls, while six studies [28,30,31,34,35,37] (86%) did not report a significant difference (Table 1). In this subset, four case–control studies (57%) provided sufficient data to calculate a pooled odds ratio of 0.82 [0.73–0.92], suggesting 18% lower odds of neuropathic outcomes in cases versus controls [28,30,32,35] (Table 2 and Figure 8).

**Figure 9:**
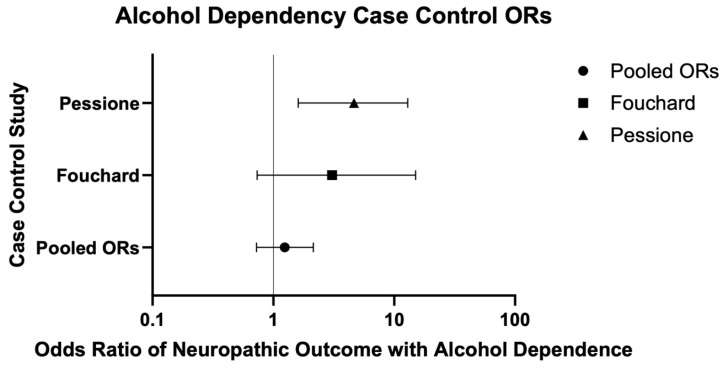
Forest plot of odds ratios of neuropathic outcome according to alcohol dependence in case–control studies [29,36].

The remaining five case–control studies assessed alcohol dependence and neuropathy/NP. Of these, four studies (80%) reported a significant positive association, either with a greater prevalence of alcohol dependence, parental history of alcoholism, and higher weekly alcohol consumption among those with neuropathy [36], longer duration of alcohol dependence, and larger total lifetime dose of alcohol [27], or with diminished electrophysiological measures of nerve function in individuals with a history of heavy alcohol consumption, including reduced sensory and motor nerve conduction velocities, amplitudes, latencies, and fiber densities [33,38]. The fifth study (20%) did not report significant findings [29]. Among these, two case–control studies (40%) provided sufficient data to calculate a pooled odds ratio of 1.24 [0.73–2.15], indicating no clear association [29,36] (Table 2 and Figure 9).

**Figure 10:**
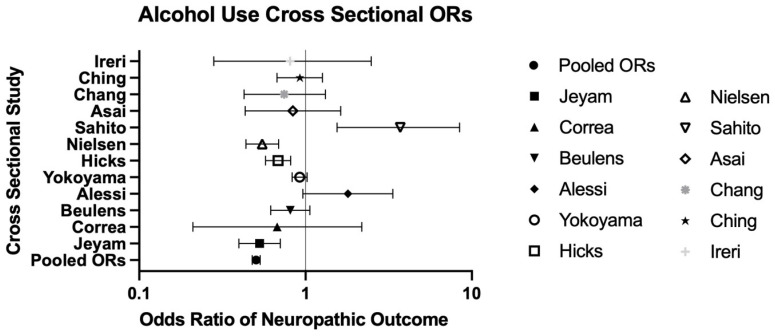
Forest plot of odds ratios of neuropathic outcome according to alcohol consumption in cross-sectional studies [39,42,43,46,48,51,57,58,59,70,73,84],.

Of the 25 cross-sectional studies reporting alcohol consumption and neuropathy/NP, ten (40%) reported a statistically significant positive association [39,54,62,63,64,68,70,73,74,81]. Neuropathy prevalence was significantly higher in individuals with high to excessive alcohol consumption, a longer history of alcohol consumption, and chronic alcoholism [39,68,70,73,74,81], with stronger associations observed in those with shorter diabetes duration and type II fiber atrophy [54,62]. Heavy alcohol consumption, defined as >4 drinks per day, and binary self-reported indicators of alcohol-related harm, including seeking professional help for alcoholism, withdrawal symptoms, and social/legal issues, were also associated with significant electrophysiological perturbations [63,64] (Table 1). In contrast, three cross-sectional studies (12%) reported a statistically significant protective association, where moderate alcohol consumption was linked to lower neuropathy prevalence [43,59], or decreased monofilament insensitivity [83], while another study (4%) found that former alcohol consumption was associated with greater neuropathy prevalence compared to current consumption [84] (Table 1). The remaining eleven cross-sectional studies (44%) did not report a statistically significant association [42,45,46,48,49,51,57,58,72,75,76]. Cumulatively, twelve studies (48%) provided sufficient data to calculate a pooled odds ratio of 0.50 [0.48–0.53], corresponding to 50% lower odds of prevalent neuropathy among those reporting alcohol exposure compared to non-use [39,42,43,46,48,51,57,58,59,70,73,84] (Table 2 and Figure 10).

**Figure 11:**
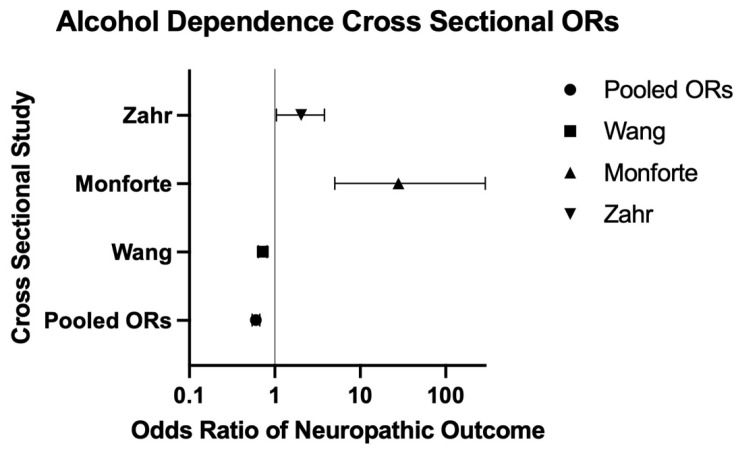
Forest plot of odds ratios of neuropathic outcome according to alcohol dependence in cross-sectional studies [67,80,85].

Of the 22 cross-sectional studies reporting alcohol dependence and neuropathy/NP, 18 (82%) reported a statistically significant positive association [40,41,44,47,50,52,53,61,66,67,69,71,78,79,82,85,86,87]. Longer durations of alcohol consumption and alcohol dependence were associated with greater neuropathy severity [47,53,79,82,86,87] and significantly diminished electrophysiological responses [40,41,44,50,52,61,66,67,69,71,78,85] (Table 1). Of the remaining four (18%) cross-sectional studies, three (14%) identified a significantly lower prevalence of alcohol dependence among individuals with neuropathy [55,60,80], while one study (5%) reported alcohol dependence in 6.36% of individuals with neuropathy but did not provide further statistics [65] (Table 1). Cumulatively, three studies provided sufficient data to calculate an odds ratio of 0.60 [0.54–0.67], suggesting that alcohol exposure is associated with 40% lower odds of prevalent neuropathy compared to non-use [67,80,85] (Table 2 and Figure 11).

**Figure 12:**
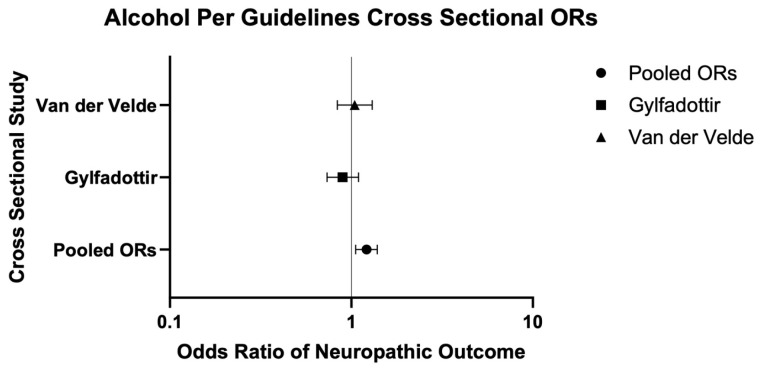
Forest plot of odds ratios of neuropathic outcome according to alcohol overconsumption as per guidelines in cross-sectional studies [56,77].

The final two cross-sectional studies assessed alcohol consumption using specific guideline-based thresholds (>14/7 units per week for males/females), and did not report a significant association individually. However, pooled analysis yielded an odds ratio of 1.21 [1.06–1.39], corresponding to 21% higher odds of prevalent neuropathy [56,77] (Table 1 and Table 2, and Figure 12).

## 4. Discussion

Findings from this systematic review suggest that varying degrees of alcohol consumption are associated with neuropathy and NP across observational study designs; however, these associations should be interpreted in the context of substantial heterogeneity in neuropathy etiologies and alcohol exposure definitions, and do not represent causal effect estimates. Over half (39/76, 51.3%) of included studies demonstrated a statistically significant positive association, whereby alcohol consumption increased the incidence, prevalence, and/or severity of neuropathy or neuropathic pain (including chemotherapy-induced PN, diabetic PoN, PN, PoN, somatic neuropathy, and UN) across a wide range of etiologies, such as diabetes, cancer, and AUD (*p* < 0.05) [12,14,17,18,19,22,27,32,33,36,38,39,40,41,44,47,50,52,53,54,61,62,63,64,66,67,68,69,70,71,73,74,78,79,81,82,85,86,87]. Conversely, 29/76 (38.2%) studies reported no association [13,16,20,21,23,24,25,26,28,29,30,31,34,35,37,42,45,46,48,49,51,56,57,58,65,72,75,76,77], while only 8/76 (10.5%) demonstrated a statistically significant negative effect (*p* < 0.05) [15,43,55,59,60,80,83,84]. Across all study designs, the overall certainty of evidence was very low, largely driven by substantial risk of bias, particularly due to reliance on self-reported alcohol exposure, as well as heterogeneity in exposure classification and outcome assessments (Figure 2, Figure 3 and Figure 4, Table 2). Despite these limitations, a significant positive association was still observed in downstream pooled analyses, consistent with the previously published literature demonstrating a high prevalence of neuropathy among those with chronic alcohol use, with estimates approaching 46%, alongside increased pain sensitivity and interference associated with heavy alcohol use and withdrawal [2,10]. Likewise, these findings align with the previously described pathophysiological mechanisms of alcohol-related neuropathy, whereby chronic and excessive alcohol use contribute to peripheral nerve injury through both direct neurotoxic effects, indirect metabolic disruptions, and potential molecular regulatory processes. Together, ethanol-induced oxidative stress and alcohol-related nutritional deficiencies may drive peripheral nervous system sensitization and degeneration, thereby explaining the increased incidence, prevalence, and severity of neuropathy and neuropathic pain observed in this review.

### 4.1. Cohort Studies

Cohort studies demonstrated the most consistent positive estimated effects of alcohol exposure on incident neuropathy outcomes pooled across all study designs, corresponding to 29% higher odds with any alcohol consumption compared to non-use [13,22,25]. These findings contrast those of the individual studies, which largely reported no significant association between alcohol consumption and neuropathic outcomes [13,20,23,25,26], or demonstrated an inverse effect [15,22]. Despite this, a subset of studies [13,25] reporting no association contributed point estimates above the null, which when pooled ultimately shifted the overall effect toward a statistically significant positive relationship (Figure 5). Due to this lack of directional concordance across studies, there was a high risk of inconsistency. Together, with a serious risk of bias, and low risk of imprecision and indirectness, the overall certainty of evidence remained very low (Table 2). However, these findings suggest that while individual studies may lack power or consistency, pooled analyses may better capture the underlying association between alcohol consumption and incident neuropathy.

Alcohol use disorder and dependence were associated with 24% higher odds of incident neuropathy [12,19,21], aligning with published studies reporting a higher risk of incident neuropathy with AUD overall [12,19], as well as improvements in electrophysiological parameters following abstinence [17,18]. Among the remaining articles, null associations may reflect either the presence of stronger competing risk factors that attenuate the detectable effect of alcohol exposure [16], or study designs in which alcohol-related risk is evaluated relative to already high-risk comparator groups, thereby limiting the ability to detect incremental effects [21]. Notably, cohort studies assessing alcohol dependence contributed some of the most robust evidence within this review, with comparatively fewer concerns related to inconsistency, indirectness, and imprecision (Table 2). However, the overall certainty of evidence remained very low due to inherent limitations of observational designs, including residual confounding and reliance on self-reported alcohol exposure, increasing the overall risk of bias.

The remaining cohort studies assessed alcohol consumption according to predefined guideline thresholds, including exceeding 14/21 units of alcohol per week for females/males [14], or consuming greater than 30 g per week irrespective of sex [24]. The findings of each individual study varied; however, the pooled effects estimate suggests a 51% increased risk of incident neuropathy/NP in those exceeding recommended guidelines versus those who are not [14,24]. The overall certainty of evidence was very low, downgraded due to moderate concerns with indirectness, reflecting arbitrary threshold-based exposure definitions, and imprecision, due to a limited number of contributing studies and wide confidence intervals (Table 2). Overall, pooled analyses across cohort studies suggest that increasing alcohol consumption and dependence are associated with a higher risk of incident neuropathy and related outcomes. However, substantial heterogeneity in underlying etiologies, exposure classification and quantification, and outcome measurement limits the generalizability of pooled effect estimates, which are often derived from restricted subsets of the available data. Likewise, although concerns related to inconsistency, indirectness, and imprecision were generally modest, the overall certainty of evidence remained very low due to a serious risk of bias from subjective outcome measures and substantial loss to follow-up (Table 2). As such, there is a clear need for more robust study designs, including those incorporating objective outcome measures, standardized and quantitative exposure assessments, and improved longitudinal follow-up, to more accurately estimate this relationship.

### 4.2. Case–Control Studies

The estimated effect of alcohol consumption on neuropathy/NP in case–control studies was highly variable. Despite the majority of studies reporting no specific association [28,30,31,34,35,37], the pooled estimate suggests that cases, or those who endorse consuming any alcohol, have 18% lower odds of neuropathic outcomes compared to controls [28,30,32,35]. Effect estimates across studies largely clustered around the null and were susceptible to unadjusted confounding [28,30,35]. Alcohol consumption was consistently assessed as a secondary exposure rather than a primary variable of interest, and robust multivariable analyses were often not performed, limiting the ability to isolate its independent effect from other lifestyle factors such as smoking, diet, and physical activity. Furthermore, the only study reporting a positive association [32] was imprecise, with wide confidence intervals, resulting in reduced weighting in the pooled analysis. Together, these factors suggest that the observed inverse pooled estimate does not reflect a true protective relationship but rather arises from residual confounding and imprecision in the contributing studies. Given the known risk of bias inherent to observational studies, along with moderate to high concerns related to inconsistency, indirectness, and imprecision, the overall certainty of evidence remained very low (Table 2).

Similarly, no clear association was identified for the effect of alcohol dependence on neuropathy/NP [29,36]. Although the majority of studies reported a statistically significant positive association [27,33,36,38], including greater peripheral neuropathy prevalence and impaired electrophysiological parameters in those with a history of heavy alcohol use or dependence, only two studies provided sufficient data for pooled analysis [29,36], one of which reported no association at all [29]. As a result, the pooled effect estimate remained inconclusive, and the limited number of contributing studies and variability in exposure and outcome assessments resulted in moderate levels of indirectness and imprecision. Despite a non-serious risk of bias and low inconsistency, the overall certainty of evidence was downgraded to very low (Table 2). As such, these findings highlight the need for more robust studies to better estimate these relationships.

### 4.3. Cross-Sectional Studies

The pooled estimates for both the effect of alcohol consumption and dependence on the prevalence of neuropathy/NP indicate a protective effect, whereby alcohol use is associated with 50%, and 40% lower prevalence of neuropathy compared to non-use, respectively [39,42,43,46,48,51,57,58,59,67,70,73,80,84,85]. This directly contradicts the majority of studies (28/47, 60%) reporting greater prevalence of PN, diabetic PN, chemotherapy-induced PN, PoN, and NP, with greater duration, and frequency of alcohol consumption and dependence [39,40,41,44,47,50,52,53,54,61,62,63,64,66,67,68,69,70,71,73,74,78,79,81,82,85,86,87]. Given the cross-sectional nature of this subset of the literature, a causal relationship cannot be established, as exposure and outcome are assessed simultaneously without accounting for temporal sequence. This enables reverse causation to influence the observed associations, specifically protopathic bias within this body of the literature, whereby individuals with neuropathy may reduce or cease alcohol consumption, resulting in a disproportionate representation of affected individuals within lower-exposure categories. Similarly, survivorship and selection effects may further distort this relationship, as individuals with more severe disease or higher levels of alcohol exposure may be less likely to participate due to physical, cognitive, or psychosocial limitations. Together, these mechanisms may reflect or exaggerate underlying U-shaped relationships, whereby moderate alcohol consumption appears protective relative to both abstinence and heavy use, while also exaggerating inverse associations in pooled analyses. The majority of studies reporting a negative and seemingly protective effect emphasize these phenomena as key limitations within their own analyses [43,59,60,84]. Given these methodological constraints, and the serious risk of bias inherent to cross-sectional designs, the overall certainty of evidence remained very low (Table 2).

Lastly, pooled estimates from the remaining cross-sectional studies examining alcohol consumption per guideline thresholds suggest that intake exceeding 7/14 units per week for females/males is associated with a 21% higher prevalence of neuropathy/NP compared to lower consumption [56,77]. However, individual study results were non-significant and given the arbitrary nature of guideline-based exposure definitions, wide confidence intervals, and the limited number of contributing studies, the relationship cannot be confidently interpreted. Accordingly, the overall certainty of evidence was very low due to these methodological constraints (Table 2). Overall, cross-sectional studies reported mixed and often discordant findings, reflecting their inherent limitations and significant heterogeneity across study designs. Reverse causation, residual confounding, and selection and survivorship bias substantially limit the interpretability of these results, and larger longitudinal studies with greater methodological rigor are needed to more accurately estimate the effect of alcohol consumption on the prevalence of neuropathy/NP.

### 4.4. Limitations

Overall, this review was limited by substantial methodological heterogeneity across all included studies, given significant variability in neuropathy and NP outcome assessments, underlying etiologies, and the classification of alcohol exposure, which implicated various binary and categorical groupings. In particular, several exposure definitions did not consistently distinguish between former, current, lifetime, or unspecified alcohol use, further limiting temporal interpretation and increasing the potential for misclassification. This significantly impacted comparability within study designs during downstream pooled analyses and limited generalizability to a broader clinical context. Alcohol use was measured exclusively by self-report, without objective biological or physiological validation, increasing susceptibility to misclassification and information bias. Given the considerable physical, cognitive, and psychosocial burden associated with alcohol dependence, significant attrition bias was likewise evident. Additionally, in several studies, alcohol consumption was treated as a secondary variable rather than the primary exposure of interest, with limited adjustment for other lifestyle factors such as diet (which may directly impact compressive neuropathies), thereby limiting the ability to accurately estimate its independent effects on neuropathic outcomes. Likewise, although studies enrolling alcohol-dependent populations or individuals with alcohol-related neuropathy were interpreted within the context of their underlying study populations and exposure definitions, their inclusion may have also obscured the independent effect of alcohol as a risk factor. Reverse causation and protopathic bias represented additional key limitations, both within the included studies and this review itself, as individuals with more severe disease may reduce or cease alcohol consumption prior to or during study participation, potentially biasing effect estimates and obscuring the true association. Lastly, several pooled estimates were derived from a limited number of studies or restricted subsets of data, reducing the precision and stability of effect estimates. Given that the included studies were observational in nature, the overall certainty of evidence was inherently limited, with risk of bias and other methodological constraints further reducing its consistency, precision, and directness. However, despite these limitations, the pooled analyses demonstrated a statistically significant positive association between alcohol exposure and neuropathy/NP incidence and severity.

## 5. Conclusions

This systematic review identified a broad body of the literature examining the association between alcohol exposure and neuropathy/NP incidence, prevalence, and severity across a range of underlying etiologies. The reported data and pooled effect estimates suggest that alcohol consumption and dependence are associated with increased odds of incident neuropathy and NP overall. These associations were most consistently observed in common non-infectious conditions, including diabetes, cancer, and alcohol use disorders, while a notable gap remains in understanding their impact within infectious etiologies such as leprosy, highlighting the need for further research in underrepresented infectious disease contexts where excessive alcohol consumption may exacerbate underlying disease states. Overall, alcohol consumption and dependence appear to directly influence neuropathic outcomes, while cessation may represent a low-risk, low-cost, and low-tech adjunctive therapeutic strategy. Although pooled estimates were directionally consistent with the broader literature, indicating increased incidence and severity of neuropathy/NP with greater alcohol consumption, the strength of these associations is limited by substantial methodological constraints.

### Future Directions

Future research should prioritize larger, well-designed studies, including comprehensive abstinence-based and/or harm reduction-based interventional trials, to strengthen the current evidence base. Standardized and well-defined alcohol exposure classifications, including clear differentiation between current, former, and lifetime use, alongside consistent neuropathy outcome measures and disease-specific analyses, will be critical to improving comparability across studies. Additionally, longitudinal designs with improved temporal resolution are needed to better address reverse causation and clarify the independent effects of alcohol on neuropathy and NP. Ultimately, such efforts will be essential to inform targeted, evidence-based strategies for reducing the burden of neuropathy and NP across diverse clinical populations.

## Figures and Tables

**Table 1:** Characteristics of all observational studies of alcohol and neuropathy/NP included in this review.

Author (Year)	Study Design	Setting	N	Sex N (F:M)	Age (Mean ± SD, (Range))	Population/Etiology	Lifestyle	Outcomes
Adler (1997) [12]	Cohort Study	US	With Ne: 58; Without Ne: 230	With Ne: 1:57; Without Ne: 11:219	With Ne: 64.0; Without Ne: 61.5	DM ± Incident Ne	Alcohol: CAGE score, history of treatment, current use	High (4) CAGE alcohol score significantly associated with incident Ne (41.7% vs. 58.3%, *p* = 0.049; β = 1.94, SE = 0.7281, 6.96 [1.67–28.99], *p* = 0.008).
Braffett (2020) [13]	Cohort Study	US	With DPN: 455; Without DPN: 931	With DPN: 182:273; Without DPN: 475:456	^ With DPN: 29 (24, 34); Without DPN 26 (21, 32)	T1DM ± DPN	Occasional or regular alcohol use	Alcohol consumption not significantly associated with DPN (1.14 [0.93–1.41], *p* > 0.05).
Christensen (2020) [14]	Cohort Study	Denmark	Overall: 5249; With DPoN: 938; With DPoN + Pain: 386	Overall: 2205:3144	^ 65 (57, 72)	T2DM ± DPoN ± Pain	Alcohol: ≤14 (F)/21 (M) units, >14 (F)/21 (M) units	Alcohol consumption above recommended limit significantly associated with increased pain prevalence (aPR: 1.31 [1.01–1.69].
Elgendy (2019) [15]	Cohort Study	Canada	1413	705:708	60 ± 8.4	T2DM ± PN	Alcohol frequency: Never, ≤1/mo, 2–4/mo, 2–3/wk, ≥4/wk; Number of drinks/d	High alcohol frequency + depressive symptoms significantly associated with prevalent Ne (1.02 [1.00–1.04], *p* = 0.04).
Ellis (2020) [16]	Cohort Study	US	254	54:200	43.5 ± 8.01	HIV ± DSPoN	Lifetime Alcohol Abuse: Yes, No	Lifetime alcohol abuse not significantly associated with DSPoN (statistics NR).
Hawley (1982) [17]	Cohort Study	US	63	0:63	53.3 (29–69)	PN due to Alcohol	History of alcoholism	Alcohol abstinence associated with significant electrophysiology improvement in PN (+0.12 m/s/abstinent mo, *p* < 0.05).
Hillbom (1984) [18]	Cohort Study	Sweden	10	0:10	53.1 (38–72)	Chronic alcoholism ± PN	History of alcoholism and seeking rehabilitation	Alcohol abstinence associated with improvement in clinical/electrophysiological PN.
Iturralde (2024) [19]	Cohort Study	US	Overall: 222,334; AUD+: 1998; AUD−: 220,336	Overall: 106,353:115,978; AUD+: 477:1521; AUD−: 105,876:114,457	AUD+: 57 ± 11.3; AUD−: 64.3 ± 12.7	T2DM ± AUD ± Ne	AUD (yes, no)	AUD significantly associated with a higher risk of Ne in unadjusted (*p* < 0.001) and adjusted (*p* < 0.001) models.
Kerns (2025) [20]	Cohort Study	US	1 drink/d: 539; >1 drink/d: 76	0:615	45 (22–79)	Testicular cancer survivors ± worsening PSN, or NP	History of alcohol consumption (1 drink/d, >1 drink/d)	Alcohol consumption (>1 drink/d) not significantly associated with PSN (0.75 [0.40–1.44], *p* = 0.392), or NP (0.76 [0.29–1.98], *p* = 0.570).
Khan (2023) [21]	Cohort Study	US	TUD: 8009; TAUD: 1672; PSUD: 642; TUD Co: 8009; TAUD Co: 1672; PSUD Co: 642	TUD: 4660:3349; TAUD: 582:1090; PSUD: 233:409; TUD Co: 4665:3344; TAUD Co: 584:1088; PSUD Co: 234:408	TUD: 61.6 ± 12.1; TAUD: 61.52 ± 10.3; PSUD: 57.84 ± 8.3; TUD Co: 61.6 ± 12.1; TAUD Co: 61.42 ± 10; PSUD Co: 57.88 ± 8.1	T2DM + Hypertension ± Ne	TUD: Yes, No; TAUD: Yes, No; PSUD: Yes, No	PSUD associated with significantly higher risk of DN (1.76 [1.33–2.32], *p* < 0.05) compared to TUD.
Kindl (2021) [22]	Cohort Study	Germany	With MSK: 255; With CRPS: 223	With MSK: 160:95; With CRPS: 173:50	With MSK: 54.6 (20–80); With CRPS: 50.9 (18–77)	CRPS or MSK, due to trauma	Alcohol Consumption: Yes, No, Daily, Weekly, Monthly	Prevalence of alcohol consumption significant with MSK (58%, *p* < 0.001), and CRPS (43%, *p* < 0.001).
Kuguyo (2024) [23]	Cohort Study	Zimbabwe	252	252:0	(26–90); ^ 50 (43–61)	Breast cancer survivors ± sensory loss (cisplatin)	History of alcohol consumption	Alcohol consumption not significantly associated with diminished electrophysiology (*p* > 0.05).
Lehtinen (1993) [24]	Cohort Study	Finland	With ND: 12; Without ND: 101	With ND: 9:3; Without ND: 46:55	With ND: 57.2 ± 4.7; Without ND: 55.4 ± 10.4	DM ± ND	Alcohol use (>30 g/wk)	Alcohol use not significantly different between ND groups (17% vs. 30%, *p* > 0.05).
Sreeram (2023) [25]	Cohort Study	US	Overall: 1034; With CIPN: 704; Without CIPN: 330	Overall: 797:237; With CIPN: 570:134; Without CIPN: 227:103	Overall: 57.1 ± 10.9 (27–79); With CIPN: 55.8 ± 10.8 (27–79); Without CIPN: 59.9 ± 10.4 (27–79)	Cancer survivors ± CIPN	Alcohol use (Past 4 wks): Yes, No	Alcohol use not significantly different between CIPN groups, or associated with CIPN prevalence (51.2% vs. 46.4%, 1.10 [0.81–1.49], *p* > 0.05).
Xu (2025) [26]	Cohort Study	China	Low-Risk Stable CIPN: 148; Mod-Risk Progressive CIPN: 145; High-Risk Progressive CIPN: 57	350:0	18–44: n = 89; 45–59: n = 179; >60: n = 82	Breast cancer survivors ± CIPN	History of harmful alcohol consumption (yes, no)	History of harmful alcohol consumption not significantly different between CIPN groups (1.4% vs. 2.1% vs. 1.8%, x^2^ = 0.225, *p* = 0.894).
Agelink (1998) [27]	Case–Control Study	Germany	Ca: 35; Co: 80	Ca: 15:20; Co: 33:47	Ca: 42.9 ± 10 (28–74); Co: 41.8 ± 14.7 (27–77)	Alcoholism ± PN & CAN vs. healthy co	Duration of alcohol dependence (yrs), mean TLDA	Duration of alcohol dependence was significantly longer (6.1 ± 3.8 vs. 16.2 ± 7.1, *p* < 0.0005) and mean TLDA was significantly larger (438 ± 342 vs. 1930 ± 1173, *p* < 0.001) with AUD PN vs. without.
Doneddu (2020) [28]	Case–Control Study	Italy	Ca: 195; Co: 195	Ca: 109:86; Co: 109:86	NR	CIDP due to any etiology and their partners	Alcohol Consumption: Yes, No	Alcohol consumption not significantly associated with CIDP (0.79 [0.50–1.24], *p* > 0.05).
Fouchard (2023) [29]	Case–Control Study	France	Overall: 323; Ca: 162; Co: 161	Overall: 192:131; Ca: 88:74; Co: 104:57	Ca: 56 ± 16; Co: 69 ± 13	Cutaneous paresthesia ± SFN via IENFD due to any etiology	Alcoholism: Yes, No	Alcohol consumption not significantly different between SFN groups (3.7% vs. 1.2%, *p* > 0.05).
Franklin (1994) [30]	Case–Control Study	US	Ca: 77; Co: 200	Ca: 29:48; Co: 118:82	Ca: 61.7; Co: 58.6	NIDDM ± DSN	Alcohol use: never, g/wk (<20, >20)	Alcohol (g/wk: <20, >20) not significantly associated with DSN (0.71 [0.29–1.72] *p* = 0.69, 1.03 [0.40–2.62]).
Frost (2013) [31]	Case–Control Study	Denmark	Ca: 324; Co: 832	Ca: 121:203; Co 317:515	Ca Smokers: 49 ± 9.7; Ca Non-Smokers: 44 ± 11.5; Co Smokers: 50 ± 9; Co Non-Smokers: 48 ± 9.9	Ca: Electroneurographically confirmed UN; Co: Without UN	Alcohol: u/wk	Alcohol consumption not significantly associated with UN (0.81 [0.44–1.48]).
Gebabo (2021) [32]	Case–Control Study	Ethiopia	Overall: 528; Ca: 264; Co: 264	Ca: 101:163; Co: 105:159	Ca: <40: 43; 40–65: 178; 65+: 43; Co: <40: 64; 40–65: 178; 65+: 17	T1DM or T2DM ± PN	Alcohol Consumption (Ever): Yes, No	Alcohol consumption significantly higher with PN vs. without (5.3% vs. 1.5%, *p* = 0.024).
Koike (2001) [33]	Case–Control Study	Japan	18	0:18	47.7 ± 10.5 (31–70)	Alcoholism PoN vs. healthy co	History of heavy alcohol consumption (100 g eth/d for >10 yrs)	History of heavy alcohol consumption significantly associated with diminished electrophysiology (*p* < 0.005).
Mitchell (1990) [34]	Case–Control Study	US	IDDM: Ca: 54, Co: 56; NIDDM: Ca: 39, Co: 65	IDDM: Ca: 31:23, Co: 35:21; NIDDM: Ca: 25:14, Co: 44:21	IDDM: Ca: 36.1, Co: 32.7; NIDDM: 59.4: 39, Co: 57.7	DM ± Ne	Alcohol: median drink-yrs	Median alcohol consumption not significantly associated with Ne in IDDM (996 vs. 485). or NIDDM (0 vs. 14).
Mondelli (2020) [35]	Case–Control Study	Italy	Ca: 220; Co: 460	Ca: 84:136; Co: 242:218	Ca: 51.7 ± 11.8; Co: 47.8 ± 12.4	Ca: UNE; Co: Upper limb complaints	Alcohol: u/wk or d	Alcohol consumption not significantly different between UNE groups (*p* = 0.463).
Pessione (1995) [36]	Case–Control Study	France	Ca: 32; Co: 58	Ca: 6:26; Co: 22:36	Ca: 49 ± 10.1; Co: 46.8 ± 9.6	Alcoholism ± PN	Alcohol: parental history of alcoholism, alcohol dependence, weekly alcohol consumption (drinks)	Alcohol-related risk factors all significantly higher in those with PN vs. without in univariate (*p* < 0.01); and multivariate *p* < 0.05) analyses.
Richardson (2016) [37]	Case–Control Study	US	Ca: 50; Co: 50	Ca: 18:32; Co: 34:16	Ca: 48.4 ± 12.8; Co: 39.2 ± 12	Ca: UNE+; Co: UNE-	Alcohol: Eth/wk (ounces)	Eth/wk did not significantly differ between UNE groups (1.2 ± 1.9 vs. 1.2 ± 2.2, *p* = 0.993).
Walsh (1970) [38]	Case–Control Study	Australia	Ca: 11; Co: 20	1:10	Ca: 58 (41–73); Co: 54 (38–73)	PN due to alcoholism	History of PN due to alcoholism: Yes, No	History of heavy alcohol consumption associated with diminished electrophysiology (*p* < 0.01), and fiber densities (3.39 ± 0.86 vs. 5.78 ± 0.90, *p* < 0.001).
Alessi (2020) [39]	Cross-Sectional Study	US	Overall: 934; Never Drinker: 103; Former Drinker: 89; Nonbinge Drinker: 567; Binge Drinker: 174	Overall: 569:365; Never Drinker: 61:42; Former Drinker: 51:38; Nonbinge Drinker: 373:194; Binge Drinker: 84:90	Overall: 38.3 ± 15.8; Never Drinker: 31.8 ± 16.8; Former Drinker: 44.1 ± 16.1; Nonbinge Drinker: 39.8 ± 15.8; Binge Drinker: 34 ± 13	T1DM ± PN	Alcohol Consumption: Never, Former, Current (Nonbinge), Current (Binge)	Ne significantly lower in never vs. former alcohol consumption (11% vs. 35%, *p* = 0.006).
Ammendola (2000) [40]	Cross-Sectional with Nested Case–Control	Italy	62	18:44	43.3 (28–69)	Chronic alcoholism (>100 g/d for >2 yrs) ± PN	Mean alcohol-related disease duration; Mean TLDE	Diminished electrophysiology significantly associated with AUD (*p* < 0.01), longer disease duration (*p* < 0.01), and higher TLDE (*p* < 0.05).
Ammendola (2001) [41]	Cross-Sectional Study	Italy	Overall: 76; With Ne: 51; Without Ne: 25	Overall: 21:55; With Ne: 14:37; Without Ne: 7:18	Overall: 24–69; With Ne: 45.3 ± 9.4; Without Ne: 39.1 ± 7.7	Chronic alcoholism ± PN	Family history of alcoholism (Yes, No); duration of alcoholism; TLDE	Prolonged alcohol-related disease duration (16.2 ± 9.4 vs. 11.1 ± 8.2, *p* < 0.05), high TLDE (27.9 vs. 14.8 ± 15.9, *p* < 0.05), and diminished electrophysiology (*p* < 0.01) significantly associated with Ne.
Asai (2022) [42]	Cross-Sectional Study	Japan	Overall: 817; With CP: 35; Without CP: 782	Overall: 431:386; With CP: 24:11; Without CP: 407:375	With CP: 63.91 [60.11–67.72]; Without CP: 63.75 [63.02–67.72]	Chronic neck/shoulder/upper limb pain due to any etiology	Current drinker: Yes, No	Alcohol consumption not significantly different between CP groups (42.86% vs. 47.19%, *p* > 0.05).
Beulens (2008) [43]	Cross-Sectional Study	Europe	1857	893:964	(15–60)	T1DM ± Ne	Alcohol consumption (g/wk)	Moderate alcohol consumption (30–70 g/wk)/frequency (5–7 d/wk) associated with significantly lower risk of Ne (0.61 [0.41–0.91], *p* < 0.01; 0.49 [0.34–0.71], *p* < 0.001).
Blackstock (1972) [44]	Cross-Sectional Study	United Kingdom	Chronic Alcoholism: 30; Hospital Personnel: 14	Chronic Alcoholism: 7:23; Hospital Personnel: NR	Chronic Alcoholism: 44.8 (21–65); Hospital Personnel: 36.6 ± 6.9	Chronic alcoholism ± PoN vs. hospital personnel	Chronic alcohol consumption/dependency	Greater electrophysiological perturbation in those with AUD vs. hospital personnel (*p* < 0.001).
Callaghan (2020) [45]	Cross-Sectional Study	US	BMI < 35 kg—Ne: 45; BMI > 35 kg—Ne: 110; BMI > 35 kg + Ne: 28	BMI < 35 kg—Ne: 37:8; BMI > 35 kg—Ne: 87:23; BMI > 35 kg + Ne: 18:10	BMI < 35 kg—Ne: 43.8 ± 12.1; BMI > 35 kg—Ne: 43.5 ± 11.2; BMI > 35 kg + Ne: 51.4 ± 9.6	Ca: BMI > 35 kg ± Ne; Co: BMI < 25 kg BMI < 35 kg—Ne; BMI > 35 kg—Ne; BMI > 35 kg + Ne	Alcohol: drinks/wk (past yr)	Alcohol consumption not significantly different between Ne groups (*p* > 0.05).
Chang (2025) [46]	Cross-Sectional Study	China	DPN+: 163; DPN−: 107	DPN+: 69:94; DPN-: 49:58	DPN+: 67 (63–70); DPN−: 65 (62–69)	Elderly T2DM ± DPN	History of alcohol consumption (yes, no)	Alcohol consumption not significantly correlated with DPN (*p* = 0.310).
Chaware (2022) [47]	Cross-Sectional Study	India	100	0:100	39.91	Chronic alcoholism ± PN	Chronic alcoholism (>60 g eth/d or >15 drinks/wk): <5 yrs, 5–15 yrs, >15 yrs	Greater severity of DPN significantly associated with longer consumption (<5 yrs: 21.8 ± 3.4; 5–15 yrs: 28.1 ± 3.7; >15 yrs: 33.7 ± 3.9; *p* = 0.001).
Ching (2024) [48]	Cross-Sectional Study	Malaysia	Overall: 1283; PN−: 943; PN+: 340	Overall: 635:648; PN−: 495:448; PN+: 140:200	40.6 ± 12.9 (18–80)	Any etiology ± PN	Alcohol consumption (yes, no)	Alcohol consumption not significantly different between PN groups (18.53% vs. 19.72%, *p* > 0.05).
Chukwubuzo (2022) [49]	Cross-Sectional Study	Nigeria	422	289:133	57.6 ± 10.1	T1DM or T2DM ± PN	Alcohol Consumption: Yes, No	Alcohol consumption not significantly associated with painful DPN (1.48 [0.74–2.98], *p* < 0.05).
Claus (1985) [50]	Cross-Sectional Study	Germany	Chronic Alcoholism: 30; Healthy Volunteers: 30	Chronic Alcoholism: 0:30; Healthy Volunteers: 2:28	Chronic Alcoholism: 38.2 ± 6.4 (26–48); Healthy Volunteers: 34.9 ± 6	Chronic alcohol use ± PoN	History of alcohol consumption (up to >400 mL/d for 5–12 yrs)	Alcohol consumption (>12 yrs) significantly correlated with higher PoN frequency (*p* = 0.007). Electrophysiology significantly impaired vs. control (*p* < 0.01 for all).
Correa (2023) [51]	Cross-Sectional Study	Brazil	Overall: 444; LLBP: 313; PNBP: 33; WP: 98	Overall: 289:155; LLBP: 188:125; PNBP: 26:7; WP: 75:23	Overall: 39.72 ± 14.68; LLBP: 37.02 ± 13.39; PNBP: 8.45 ± 14.30; WP: 48.78 ± 15.59	Chronic BP due to any etiology	Alcohol Abuse: Yes, No	Reported alcohol consumption: LLBP: 12.1%, PNBP: 9.1%, WP: 12.2% (statistics NR).
D’Amour (1991) [52]	Cross-Sectional Study	Canada	Chronic Alcoholism: 20; Hospital Personnel: 20	NR	Chronic Alcoholism: 46 (31–67); Hospital Personnel: 38 (21–50)	Chronic alcoholism ± PN vs. hospital personnel	History of alcohol consumption (>10 yrs or >100 g/d)	PN in 75% of AUD. Electrophysiology significantly reduced in patients with alcoholism vs. healthy hospital personnel (*p* < 0.05).
Estruch (1993) [53]	Cross-Sectional Study	Spain	Chronic Alcoholism: 250; Healthy Volunteers: 100	Chronic Alcoholism: 0:250; Healthy Volunteers: 0:100	Chronic Alcoholism: 41 ± 11 (20–65); Healthy Volunteers: 40 ± 10 (20–65)	Chronic alcoholism ± PN vs. healthy volunteers	Daily ethanol consumption (>100 g) over last 2 yrs	PN present in 16% (41) of AUD. AUD + PN had higher ethanol consumption vs. those without PN (34.7 vs. 22.4, *p* < 0.001); TLDE was an independent risk factor for PN in multivariate analysis (*p* < 0.001).
Fernandez-Sola (1995) [54]	Cross-Sectional Study	Spain	100	0:100	41 ± 9 (25–60)	Chronic alcoholism ± PN	Daily eth intake (g/d); duration of eth consumption; TLDE	PN (39% vs. 15%, *p* = 0.014) and TLDE (31.7 ± 17 vs. 23.3 ± 14, *p* = 0.01) significantly higher in those with chronic alcoholism + type II fiber atrophy vs. without.
Gierthmühlen (2024) [55]	Cross-Sectional Study	Denmark, France, Germany, Israel, Spain, UK	Overall: 1181; Ne + Pain: 843; Ne − Pain: 338	Overall: 405:776; Ne + Pain: 285:558; Ne − Pain: 120:218	Overall: 65.8 ± 12 (19–92); Ne + Pain: 64.9 ± 12.4 (19–92); Ne − Pain: 68.1 ± 10.6 (19–87)	Any etiology Ne ± pain	Current or previous alcohol misuse (yes, no)	Prevalence of current or previous alcohol misuse significantly lower among those with pain vs. without (6.6% vs. 11.5%, *p* = 0.0022).
Gylfadottir (2020) [56]	Cross-Sectional Study	Denmark	5514	2355:3159	64.1 ± 10.9	T2DM ± DPoN	Alcohol: >7 (F)/14 (M) units	Alcohol consumption above recommended limit not significantly associated with DPoN (0.94 [0.74–1.18], *p* > 0.05), or painful DPoN (1.09 [0.81–1.46], *p* > 0.05), in multivariable logistic regression.
Hicks (2022) [57]	Cross-Sectional Study	US	Overall: 6902; With PN: 1181; Without PN: 5721	Overall: 3589:3313; With PN: 443:738; Without PN: 3101:2620	% (!) 40–49: 36 (0.9); 50–59: 27.8 (0.8); 60–69: 18.2 (0.6); 70–79: 12.8 (0.4); ≥80: 5.2 (0.3)	DM ± PN	Alcohol: Never, Former, Current	Alcohol consumption reported between PN groups: Never: 16.4%, Former: 27.2%, Current: 56.5% vs. 11.8%, 20.9%, 67.3% (statistics NR).
Ireri (2024) [58]	Cross-Sectional Study	Kenya	314	182:132	58.49 ± 17.43	DM ± Ne	History of alcohol consumption	Alcohol consumption not significantly associated with Ne (*p* = 0.481).
Jeyam (2020) [59]	Cross-Sectional Study	Scotland	Overall: 5558; With DPN 715; Without DPN 4842	Overall: 2449:3109; With DPN: 320:395; Without DPN 2129:2713	^ Overall: 44.7 (33, 55.2); With DPN: 50.6 (41, 59.3); Without DPN: 43.7 (32, 54.4)	T1DM ± DPN	Alcohol (u/wk): 2–6, 6–14, 14–21, 21–32, >32	Alcohol consumption below 32 u/wk associated with lower odds of DPN (0.47 [0.29–0.75], *p* < 0.05), while above 32 u/wk was not (0.88 [0.56–1.38], *p* > 0.05). Authors suggest reverse causation–protopathic bias.
Li (2023) [60]	Cross-Sectional Study	China	Overall: 25,710; With PDPN: 14,699; Without PDPN: 11,011	Overall: 10,785:14,925; With PDPN: 6240:8459; Without PDPN: 4545:6466	^ Overall: 63 (55, 71); With PDPN: 65 (56, 73); Without PDPN: 61 (53, 69)	T2DM ± PDPN	Alcohol Abuse: Yes, No	PDPN significantly lower with alcohol abuse vs. without (54.1% vs. 57.1%, *p* = 0.002). Authors suggest reverse causation–protopathic bias.
Mawdsley & Mayer (1965) [61]	Cross-Sectional Study	US	Chronic Alcoholism: 76; Healthy Personnel: 105	Chronic Alcoholism: 12:64; Healthy Personnel: NR	Chronic Alcoholism: 25–69; Healthy Personnel: 20–70	Chronic alcohol use ± PN	History of alcohol consumption (1 pint whisky to 1 gallon wine/d)	Electrophysiology significantly diminished vs. control (*p* < 0.001 for all).
McCulloch (1980) [62]	Cross-Sectional	Scotland	541	0:541	44 ± 11.6 (20–59)	DM ± PN	Alcohol Intake: Moderate (6 drinks/yr to 10 drinks/wk) vs. Excessive (3–4 drinks/d to diagnosed alcoholism)	PN significantly higher in DM men with excessive alcohol consumption vs. those with moderate (36% vs. 14%, *p* < 0.001) and most evident with shorter durations of DM (<5 yrs: 32% vs. 8%, *p* < 0.001).
Melgaard (1986) [63]	Cross-Sectional Study	Denmark	468	0:468	45	“Normal” population ± PoN	Alcohol questionnaire including duration and frequency of use	Dependence-related behaviors were significantly correlated with disturbed electrophysiology (*p* < 0.05).
Mellion (2014) [64]	Cross-Sectional Study	US	Overall: 18; Heavy Drinkers: 9; Healthy Co: 9	9:9	Heavy Drinkers: 35.4; Healthy Co: 43.3	Heavy alcohol drinking (>5 drinks/d (M), >4 drinks/d (F)) + PN vs. healthy co	Eth consumed daily (g); Average duration heavy drinking	Individuals with a history of heavy alcohol consumption exhibited diminished electrophysiology, compared to those without (*p* < 0.05).
Mick (2020) [65]	Cross-Sectional Study	France, Italy, Spain, UK	1030	651:379	60.2 ± 15.32; ^ 61 (49–72)	Localized NP due to any etiology	Alcohol Abuse/Dependence: Current, Past, Never	Current or past alcohol dependence/abuse was reported in 6.36% of patients with NP (further statistics NR).
Mills (1986) [66]	Cross-Sectional Study	UK	19	4:15	(30–71)	Chronic alcoholism ± PN	History of alcoholism (120 g eth/d for 4+ yrs)	Chronic alcohol consumption (>100 g/d) significantly associated with electrophysiological PN in 12/19 (statistics NR).
Monforte (1995) [67]	Cross-Sectional Study	Spain	Alcohol-Dependent: 107; Healthy Reference: 61	Alcohol-Dependent: 18:89; Healthy Reference: 10:51	Alcohol-Dependent: 43 ± 11; Healthy Reference: 41 ± 14	Chronic alcohol use ± PN	History of alcohol consumption (>100 g/d (M) or >80 g/d (F) for >2 yrs)	Electrophysiological PN identified in 36.8% of alcohol-dependent individuals vs. 1 control (1.64%), *p* < 0.001). Diminished electrophysiology correlated with greater TLDE (r = −0.43, *p* < 0.001).
Nagakura (2023) [68]	Ecological Cross-Sectional Study	Japan	Pregabalin Reimbursement Claims per 1000 population; up to 126 million	NR	(40–74)	NP due to any etiology treated with pregabalin	Alcohol: Daily, Sometimes, Rarely/Never	Excessive alcohol consumption significantly associated with prevalent NP (β = 0.2683, *p* < 0.01); low-to-moderate intake reduced prevalence (r = −0.4713, *p* < 0.01); daily intake increased prevalence (r = 0.6253, *p* < 0.01).
Nicolosi (2005) [69]	Cross-Sectional Study	Italy	40	4:36	49.2 ± 10.3 (33–76)	Chronic alcohol use ± SoN	History of alcohol consumption (100–400 g/d for 5–25 yrs)	SoN in 62.5% (25). Electromyography scores were statistically significantly positively correlated with TLDE (r = 0.35, *p* < 0.03).
Nielsen (2022) [70]	Cross-Sectional Study	Denmark	2839	High CIPN Score: 274:146; Low CIPN Score: 1193:870	^^ High CIPN Score: 69; Low CIPN Score: 67; (18–99)	Cancer diagnosis at any stage of treatment ± CIPN	Alcohol: yes/no + u/wk	Alcohol consumption significantly different between CIPN groups (60.2% vs. 73.5%, *p* < 0.001); high consumption (>14 u/wk) significantly associated with high CIPN20 scores in males (22% vs. 11%, *p* = 0.002).
Papantoniou (2024) [71]	Cross-Sectional Study	Greece	90	34:56	51.98 ± 8.86 (27–74)	AUD ± PN	Duration of Abuse; Total Alcohol Units (kg alcohol/lifetime)	Alcohol abuse significantly longer in those with Ne vs. without (8.08 ± 7.25 vs. 6.92 ± 4.38, *p* = 0.024), and in those with impaired electrophysiology (*p* = 0.02).
Revesz (2022) [72]	Cross-Sectional Study	The Netherlands	Overall: 1516; With PN: 980; Without PN: 536	Overall: 634:882; With PN: 445:535; Without PN: 189:347	Overall: 69.1 ± 9.4; With PN: 70.1 ± 9.4; Without PN: 67.2 ± 9.2	Colorectal cancer survivors ± PN	Alcohol Consumption: Drinks/wk	Alcohol intake significantly lower in individuals with PN vs. without (median 4 vs. 5 drinks/wk, range 0–100 vs. 0–50, *p* = 0.006) in univariate analysis only.
Sahito (2022) [73]	Cross-Sectional Study	Pakistan	Overall: 1057; With PN: 607; Without PN: 450	Overall: 414:643; With PN: 230:377; Without PN: 184:266	30–40: 119; 41–50: 316; 51–60: 324; 61–70: 165; >70 yrs: 133	T2DM ± PN	History of alcohol intake: Yes, No	Alcohol intake reported between PN groups: 4.7% vs. 1.4% (statistics NR).
Shetty (2025) [74]	Cross-Sectional Study	India	110	9:101	41.5 ± 11.9 (22–84)	Chronic liver disease ± PN	History of alcohol consumption (≥5 yrs)	Duration (13.9 ± 6.1 yrs vs. 9.6 ± 4.6 yrs, *p* < 0.05), and quantity of alcohol consumption significantly higher in those with PN vs. without.
Srivastava (2022) [75]	Cross-Sectional Study	India	98	79:19	51.63 ± 10.68	Cancer survivors ± CIPN	History of alcohol consumption: Yes (current, former), No	History of alcohol consumption not significantly associated with CIPN sensory/motor severity (18.37% vs. 81.63%, *p* > 0.05).
Trendowski (2021) [76]	Cross-Sectional Study	US	With CIPN: 550; Without CIPN: 495	With CIPN: 440:110; Without CIPN: 355:140	^^ With CIPN: 56 (23–79); Without CIPN: 58 (21–79)	African American cancer survivors ± CIPN	Alcohol: yes (previous 4 wks)/no; Alcohol Consumed (drinks/wk): 0–4, ≥5	Alcohol consumption, drinks/d, not significantly associated with CIPN (aPR: 0.88 [0.68–1.14], *p* = 0.32; aPR: 0.99 [0.91–1.07], *p* = 0.73).
Van der Velde (2020) [77]	Cross-Sectional Study	The Netherlands	Overall: 2401; High Sural SNAPA: 793; Med Sural SNAPA: 796; Low Sural SNAPA: 812	Overall: 1174:1227; High Sural SNAPA: 464:329; Med Sural SNAPA: 377:419; Low Sural SNAPA: 334:478	Overall: 59.3 ± 8.2; High Sural SNAPA: 56.4 ± 8.2; Med Sural SNAPA: 59.4 ± 7.9; Low Sural SNAPA: 62 ± 7.5	T2DM ± PN	Alcohol: >7 (F)/14 (M) units	Alcohol consumption reported between PN groups: High: 26.7%, Medium: 25.8%, Low: 27.6% (statistics NR).
Villalta (1989) [78]	Cross-Sectional Study	Spain	Chronic Alcoholism: 70; Hospital Personnel: 70	Chronic Alcoholism: 0:70; Hospital Personnel: NR	Chronic Alcoholism: 39.7 ± 9.6 (20–60); Hospital Personnel: 39.2 ± 11 (20–59)	Chronic alcoholism + PN vs. hospital personnel	History of alcohol consumption (>100 g/d for >2 yrs)	Prevalence of PN in 37% (21/56). Motor CV significantly negatively correlated with TLDE (r = −0.28, *p* < 0.05).
Vittadini (2001) [79]	Cross-Sectional Study	Italy	296	87:209	45.2 ± 11.2 (20–77)	Chronic alcohol use ± PoN	History of alcohol consumption (>100 g/d)	Prevalence of electrophysiological PoN in 48.6% (144/296). PoN severity increased as duration of alcohol misuse lengthened (19.8% at 5 yrs to 40.4% at 10 yrs, *p* < 0.05).
Wang (2023) [80]	Cross-Sectional Study	China	Overall: 14,908; With DPN: 10,084; Without DPN: 4824	Overall: 6322:8586; With DPN: 4365:5719; Without DPN: 1957:2867	Overall: 61.3 ± 13, ^ 62 (53, 70); With DPN: 62.6 ± 12.5, ^ 63 (55, 71); Without DPN: 58.5 ± 13.5, ^ 59 (50, 67)	T2DM ± DPN	Alcohol Abuse (Pure Consumption in g/d): >40 (F)/60 (M); Never, Former, Current	Alcohol abuse significantly lower among those with DPN vs. without (8.2% vs. 11%, *p* < 0.001).
Wetterling (1999) [81]	Cross-Sectional Study	Germany	241	64:177	F: 43.8 ± 8.8; M: 41 ± 9.9	Chronic alcohol use ± PoN	History of alcohol consumption: episodic (<1 d/wk), frequent (>3 d/wk), continuous (daily)	Prevalence of PoN increased with increasing alcohol consumption (11.3%, 29.6%, 29.9%, *p* = 0.025), and TLDE (*p* = 0.0104).
Wilson & Thompson (2021) [82]	Cross-Sectional Study	UK	Detoxification+: 17; Detoxification−: 13	Detoxification+: 6:11; Detoxification−: 6:7	Detoxification+: 46.8 ± 9 (37–64); Detoxification−: 54.5 ± 10.5 (30–70)	High-risk alcohol use ± history of detoxification ± NP	High-risk alcohol use via FAST score ≥ 3	Prevalence of NP (IDPQ ≥ 3) significantly greater in those with history of detoxification (8/9) vs. those without (1/12), *p* = 0.04.
Wu (2025) [83]	Cross-Sectional Study	US	Overall: 1068; MNSI+: 666; MNSI−: 402	641:427	84.1 (78–100)	Very old adults ± PN	Alcohol: Never, Former, Current	Current, former alcohol use not associated with MNSI-defined neuropathy (0.90 [0.59–1.37], 1.02 [0.67–1.54], respectively). Former use not associated with monofilament insensitivity (0.78 [0.52–1.16]), whereas current use was (0.65 [0.43–0.98]).
Yokoyama (2020) [84]	Cross-Sectional Study	Japan	Overall: 9914; Without DPoN: 6180; With DPoN: 2745 (with DPoNS: 1689; with UDoPN: 989)	Overall: 3715:6139; Without DPoN: 2273:3904; With DPoN: 1041:1705 (with DPoNS: 664:1025, with UDPoN: 397:530)	^^ Overall: 66 (69–73); Without DPoN: 65 (57–71); With DPoN: 70 (63–77) (with DPoNS: 69 (63–76), with UDPoN: 67 (59–75))	T2DM ± DPoN	Alcohol: Current, Former, Never	Former alcohol consumption associated with higher odds of DPN (2.02 [1.25–3.27], *p* = 0.004), while current was not. Authors suggest reverse causation–protopathic bias.
Zahr (2019) [85]	Cross-Sectional Study	US	Ca: 154; Co: 99	Ca: 43:111; Co: 41:58	Ca: 49.8 ± 10.5 (21–77); Co: 50.9 ± 13.3 (21–74)	Ca: AUD ± PN; Co: healthy ± PN	History of AUD	Prevalence of PN (Ca: 20 vs. 134; Co: 3 vs. 96; *p* = 0.007), vibration perception impairment (Ca: 41 vs. 113; Co: 15 vs. 84; *p* = 0.03), greater with history of AUD.
Zambelis (2005) [86]	Cross-Sectional Study	Greece	98	22:17	45.2 ± 9.9 (27–70)	Chronic alcohol dependence ± PoN	Daily alcohol consumption (g); duration of alcohol abuse	Duration of alcohol consumption significantly higher in those with PoN vs. without (19.19 vs. 14.14, *p* = 0.03).
Zambelis (2016) [87]	Cross-Sectional Study	Greece	99	23:76	47 ± 19.6	Alcohol dependence ± Ulnar Ne	Duration of alcohol dependence	UNE significantly associated with duration of alcohol dependence (1.09 [1.04–1.15], *p* < 0.001).

**APA:** action potential amplitude; **aPR:** adjusted prevalence ratio; **aRR:** adjusted risk ratio; **AUD:** alcohol use disorder; **BMI:** body mass index; **BP:** back pain; **Ca:** cases; **CAN:** cardiovascular autonomic neuropathy; **CIDP:** chronic inflammatory demyelinating polyradiculoneuropathy; **CIPN:** chemotherapy-induced peripheral neuropathy; **CIPN20:** European Organization for Research and Treatment of Cancer CIPN 20-item scale; **Co:** controls; **CP:** chronic pain; **CRPS:** complex regional pain syndrome; **CV:** conduction velocity; **d:** day; **DL:** distal latency; **DM:** diabetes mellitus; **DPN:** diabetic peripheral neuropathy; **DPoN:** diabetic polyneuropathy; **DPoNS:** diabetic polyneuropathy-related sensory symptoms/signs; **DSN:** distal symmetric neuropathy; **DSPoN:** distal sensory polyneuropathy; **eth:** ethanol; **F:** female; **FAST:** Fast Alcohol Screening Tests; **g:** grams; **IDDM:** insulin-dependent diabetes mellitus; **IDPQ:** Identification of Pain Questionnaire; **IENFD:** intraepidermal nerve fiber density; **kg:** kilogram; **LLBP:** localized lower back pain; **M:** males; **mL:** milliliter; **MNSI:** Michigan Neuropathy Screening Instrument; **mo:** month; **m/s:** meters per second; **MSK:** musculoskeletal pain; **Ne:** neuropathy; **ND:** neurophysiologically deteriorated; **NIDDM:** non-insulin-dependent diabetes mellitus; **NP:** neuropathic pain; **NR:** not reported; **PDPN:** painful diabetic peripheral neuropathy; **PN:** peripheral neuropathy; **PNBP:** peripheral neuropathic back pain; **PoN:** polyneuropathy; **PSN:** peripheral sensory neuropathy; **PSUD:** polysubstance use disorder group (tobacco, alcohol, +1 other); **r:** Pearson’s correlation; **SE:** standard error; **SEP:** sensory evoked potential; **SFN:** small fiber neuropathy; **SNAPA:** sensory nerve action potential amplitude; **SoN:** somatic neuropathy; **T1DM:** type 1 diabetes mellitus; **T2DM:** type 2 diabetes mellitus; **TAUD:** tobacco and alcohol use disorder group; **TLDA/E:** total lifetime dose of alcohol/ethanol (always reported as kg alcohol/ethanol/kg body weight unless otherwise specified); **TUD:** tobacco use disorder group; **u:** units; **UDPoN:** unknown status diabetic polyneuropathy; **UN:** ulnar neuropathy; **UNE:** ulnar neuropathy at the elbow; **VPT:** vibration perception threshold; **wk:** week; **WP:** widespread pain; **x^2^**: Chi-Square statistic; **yr(s):** year(s); **β:** beta coefficient; **μV:** microvolts; **^:** median (interquartile range); **^^:** median (range); **!:** standard error; all data reported as mean ± SD, mean (range), or mean [95% CI]; outcome data reported as cases vs. controls, or as “with Ne” vs. “without Ne”, with OR [95% CI], *p*-value (adj *p*-value when available) unless otherwise specified. Disease duration always reported in years, unless otherwise specified.

**Table 2:** Summary of findings.

**Incidence of Neuropathy with/without Alcohol Consumption**											
**Population(s):** Type 1 diabetes mellitus ± diabetic peripheral neuropathy; type 1 diabetes mellitus ± neuropathy; complex regional pain syndrome and/or musculoskeletal pain via trauma; cancer survivors ± chemotherapy-induced peripheral neuropathy; type 2 diabetes mellitus + hypertension ± neuropathy; diabetes mellitus ± incident neuropathy; type 2 diabetes mellitus ± diabetic polyneuropathy ± pain; diabetes mellitus ± neurophysiologically deteriorated **Intervention:** Alcohol **Comparison:** No alcohol **Outcome:** Neuropathy incidence **Setting:** Denmark, Finland, Germany, US **Study Design:** Cohort Studies											
**Stratification**	**No. of studies**	**Neuropathy Positive (%)**	**Neuropathy Negative (%)**	**Odds ratio** **(95% CI)**	**Relative risk** **(95% CI)**	**Risk of bias**	**Inc**	**Ind**	**Imp**	**Certainty of evidence (GRADE)**	**References**
Consumed Alcohol	3	483/1369 (35.28%)	451/1515 (29.77%)	1.29 (1.10–1.50)	1.14 (1.05–1.23)	Serious	High risk	Low risk	Low risk	Very Low ⨁**◯◯◯**	Braffett (2020) [13], Kindl (2021) [22], Sreeram (2023) [25]
Alcohol Dependency	3	1054/49,327 (2.14%)	3306/191,627 (1.73%)	1.24 (1.16–1.33)	1.19 (1.12–1.25)	Serious	Mod risk	Low risk	Low risk	Very Low ⨁**◯◯◯**	Adler (1997) [12], Iturralde (2024) [19], Khan (2023) [21]
Overconsumption by Guidelines	2	354/398 (88.94%)	550/653 (84.23%)	1.51 (1.04–2.2)	1.31 (1.02–1.71)	Serious	Low risk	Mod risk	Mod risk	Very Low ⨁**◯◯◯**	Christensen (2020) [14], Lehtinen (1993) [24]
**Association between Neuropathy and Alcohol Consumption**											
**Population(s):** Diabetes mellitus ± peripheral neuropathy; chronic inflammatory demyelinating polyradiculoneuropathy; ulnar neuropathy at the elbow; non-insulin-dependent diabetes mellitus ± distal symmetric neuropathy; paresthesia ± small fiber neuropathy; alcoholism ± peripheral neuropathy; healthy controls **Intervention:** Alcohol **Comparison:** No alcohol **Outcome:** Neuropathy association **Setting:** Ethiopia, France, Italy, US **Study Design:** Case–Control Studies											
**Stratification**	**No. of studies**	**Neuropathy Positive (%)**	**Neuropathy Negative (%)**	**Odds ratio** **(95% CI)**	**Relative risk** **(95% CI)**	**Risk of bias**	**Inc**	**Ind**	**Imp**	**Certainty of evidence (GRADE)**	**References**
Consumed Alcohol	4	271/756 (35.85%)	487/1117 (43.60%)	0.82 (0.73–0.92)	---	Serious	Mod risk	High risk	Mod risk	Very Low ⨁**◯◯◯**	Gebabo (2021) [32], Doneddu (2020) [28], Franklin (1994) [30], Mondelli (2020) [35]
Alcohol Dependency	2	32/194 (16.49%)	30/219 (13.70%)	1.24 (0.73–2.15)	---	Not Serious	Low risk	Mod risk	High risk	Very Low ⨁**◯◯◯**	Fouchard (2023) [29], Pessione (1995) [36]
**Prevalence of Neuropathy with/without Alcohol Consumption**											
**Population(s):** Alcohol use disorder ± peripheral neuropathy; diabetes mellitus ± neuropathy and/or peripheral neuropathy; chronic back pain; type 1 diabetes mellitus ± neuropathy; type 2 diabetes mellitus ± diabetic peripheral neuropathy; diabetes mellitus ± diabetic polyneuropathy; chemotherapy-induced peripheral neuropathy; chronic neck/shoulder/upper limb pain **Intervention:** Alcohol **Comparison:** No alcohol **Outcome:** Neuropathy prevalence **Setting:** Brazil, China, Denmark, Europe, Japan, Kenya, Malaysia, Netherlands, Pakistan, Scotland, US **Study Design:** Cross-Sectional Studies											
**Stratification**	**No. of studies**	**Neuropathy Positive (%)**	**Neuropathy Negative (%)**	**Odds ratio** **(95% CI)**	**Relative risk ** **(95% CI)**	**Risk of bias**	**Inc**	**Ind**	**Imp**	**Certainty of evidence (GRADE)**	**References**
Consumed Alcohol	12	3548/6789 (52.26%)	15,261/22,298 (68.44%)	0.50 (0.48–0.53)	---	Serious	Mod risk	Mod risk	Low risk	Very Low ⨁**◯◯◯**	Alessi (2020) [39], Asai (2022) [42], Beulens (2008) [43], Chang (2025) [46], Ching (2024) [48], Correa (2023) [51], Hicks (2022) [57], Ireri (2024) [58], Jeyam (2020) [59], Nielsen (2022) [70], Sahito (2022) [73], Yokoyama (2020) [84]
Alcohol Dependency	3	899/10,175 (8.84%)	716/5154 (13.89%)	0.60 (0.54–0.67)	---	Serious	High risk	Low risk	Low risk	Very Low ⨁**◯◯◯**	Monforte (1995) [67], Wang (2023) [80], Zahr (2019) [85]
Overconsumption by Guidelines	2	358/1728 (20.72%)	896/5052 (17.74%)	1.21 (1.06–1.39)	---	Serious	Low risk	Mod risk	Low risk	Very Low ⨁**◯◯◯**	Gylfadottir (2020) [56], Velde (2020) [77]

**CI:** confidence interval; **Imp:** imprecision; **Inc:** inconsistency; **Ind:** indirectness; **No.:** number; **GRADE Working Group grades of evidence: High certainty** (we are very confident that the true effect lies close to that of the estimate of the effect); **moderate certainty** (we are moderately confident in the effect estimate—the true effect is likely to be close to the estimate of the effect, but there is a possibility that it is substantially different); **low certainty** (our confidence in the effect estimate is limited—the true effect may be substantially different from the estimate of the effect); **very low certainty** (we have very little confidence in the effect estimate—the true effect is likely to be substantially different from the estimate of effect).

## Data Availability

The raw data supporting the conclusions of this article will be made available by the authors on request.

## Supplementary Materials

The following supporting information can be downloaded at: https://www.mdpi.com/article/10.3390/brainsci16060551/s1, Table S1: PRISMA Checklist. Reference [88] is cited in the supplementary materials.

## Abbreviations

The following abbreviations are used in this manuscript:

AUD	Alcohol Use Disorder
BMI	Body Mass Index
CIDP	Chronic Inflammatory Demyelinating Polyradiculoneuropathy
CRPS	Complex Regional Pain Syndrome
DSN	Distal Symmetric Neuropathy
NP	Neuropathic Pain
PN	Peripheral Neuropathy
PoN	Polyneuropathy
SFN	Small Fiber Neuropathy
